# Quantitative Proteomic Analysis of Macrophages Infected with *Trypanosoma cruzi* Reveals Different Responses Dependent on the SLAMF1 Receptor and the Parasite Strain

**DOI:** 10.3390/ijms25137493

**Published:** 2024-07-08

**Authors:** Alfonso Herreros-Cabello, Javier del Moral-Salmoral, Esperanza Morato, Anabel Marina, Beatriz Barrocal, Manuel Fresno, Núria Gironès

**Affiliations:** 1 Departamento de Biología Molecular, Universidad Autónoma de Madrid (UAM), 28049 Madrid, Spain; 2 Centro de Biología Molecular Severo Ochoa (CBMSO), Consejo Superior de Investigaciones Científicas-Universidad Autónoma de Madrid (CSIC-UAM), 28049 Madrid, Spain; 3 Unidad de Proteómica, Centro de Biología Molecular Severo Ochoa (CSIC-UAM), 28049 Madrid, Spain; emorato@cbm.csic.es (E.M.);; 4 Unidad de Técnicas Bioanalíticas (BAT), Instituto de Investigación de Ciencias de la Alimentación (CIAL), Consejo Superior de Investigaciones Científicas-Universidad Autónoma de Madrid (CSIC-UAM), 28049 Madrid, Spain; 5 Instituto Universitario de Biología Molecular, Universidad Autónoma de Madrid (IUBM-UAM), 28049 Madrid, Spain; 6 Instituto de Investigación Sanitaria, Hospital Universitario de La Princesa, 28006 Madrid, Spain

**Keywords:** *Trypanosoma cruzi*, SLAMF1, Y strain, VFRA strain, quantitative proteomics

## Abstract

Chagas disease is caused by the intracellular protozoan parasite *Trypanosoma cruzi*. This disease affects mainly rural areas in Central and South America, where the insect vector is endemic. However, this disease has become a world health problem since migration has spread it to other continents. It is a complex disease with many reservoirs and vectors and high genetic variability. One of the host proteins involved in the pathogenesis is SLAMF1. This immune receptor acts during the infection of macrophages controlling parasite replication and thus affecting survival in mice but in a parasite strain-dependent manner. Therefore, we studied the role of SLAMF1 by quantitative proteomics in a macrophage in vitro infection and the different responses between Y and VFRA strains of *Trypanosoma cruzi*. We detected different significant up- or downregulated proteins involved in immune regulation processes, which are SLAMF1 and/or strain-dependent. Furthermore, independently of SLAMF1, this parasite induces different responses in macrophages to counteract the infection and kill the parasite, such as type I and II IFN responses, NLRP3 inflammasome activation, IL-18 production, TLR7 and TLR9 activation specifically with the Y strain, and IL-11 signaling specifically with the VFRA strain. These results have opened new research fields to elucidate the concrete role of SLAMF1 and discover new potential therapeutic approaches for Chagas disease.

## 1. Introduction

Chagas disease, also known as American trypanosomiasis, is a potentially life-threatening illness caused by the parasite *Trypanosoma cruzi* (*T. cruzi*), an intracellular protist that belongs to the *Kinetoplastidae* order and *Trypanosomatidae* family. This disease is named in honor of Carlos Ribeiro Justiniano Chagas, the researcher who discovered it in 1909, although the first evidence in human tissues dates to nearly 9 thousand years ago [1]. According to the World Health Organization, about 6–7 million people worldwide are estimated to be infected with *T. cruzi*, and 25 million are at risk of infection [2]. The disease is endemic to 21 countries in Central and South America, causing around 10–14 thousand deaths per year. However, Chagas disease has spread to other continents due to migration, becoming a world health problem [3,4,5]. More than 90% of patients live in the largest economies in the Western Hemisphere: Argentina, Brazil, Mexico, and the United States [6].

The immune response of mammal hosts is characterized by three principal mechanisms: recognition and killing of the parasite by the innate immune system (macrophages and dendritic cells), activation of the antigen-presenting cells, and the detection and elimination of the infected cells by the CD8+ T cells [7]. At the beginning of infection, macrophages, natural killer (NK) cells, and Tγδ cells are the first line of defense. They recognize surface molecules of *T. cruzi* by TLRs and other receptors and regulate the parasite’s replication through the generation of anti-microbial agents, such as reactive oxygen species (ROS) and reactive nitrogen intermediates (RNI) [8,9]. Furthermore, this ROS and RNI production activates the release of cytokines such as interleukin (IL)-12, tumor necrosis factor (TNF)-α, and interferon (IFN)-γ [10]. Later, in adaptive immunity, the T helper 1 lymphocytes (Th1) activate the phagocytic cells to reduce the levels of parasites. Also, Th1 cells are critical for CD8+ T cells activation and the generation of antibodies, and their inhibition can lead to uncontrolled levels of parasites and death [10].

The Signaling Lymphocytic Activation Receptor (SLAM) family is a group of type I transmembrane glycoproteins that are expressed in many hematopoietic cells such as B cells, T cells, macrophages, dendritic cells, and NK cells. The SLAM family has nine members in humans and mice [11]. Seven are self-ligand receptors, and SLAMF2 and SLAMF4 interact between them [12]. SLAMF1 (CD150) is one of the better-studied receptors of this family and is expressed in the surface of T cells, B cells, macrophages, and dendritic cells. In the adaptive and innate immune response, SLAMF1 has four principal functions: it leads to an increase in IL-4 secretion in an SAP-dependent manner following TCR activation of CD4+ T cells, while in the thymus the SLAMF1–SLAMF1 interactions between DP thymocytes promote the development of NKT cells; also, SLAMF1 interacts with the hemagglutinin of the measles virus in activated T cells, and it is a sensor of Gram-negative bacteria such as *Escherichia coli*. In this last case, SLAMF1 internalizes into the macrophage phagosome to enhance the phagocytosis of the bacteria and increase the formation and activation of the NADPH oxidase 2 (NOX2) complex [11].

More importantly, SLAMF1 is related to Chagas disease. Previous studies have identified the principal changes in infected macrophages and organs mainly at the mRNA level of several genes [13,14,15]. *Slamf1^-/-^* mice infected by the Y strain were completely protected from an acute lethal parasite challenge [13]. Also, the cardiac damage and the parasite load in the heart were reduced, suggesting that this receptor controls the susceptibility to *T. cruzi* infection, at least with the virulent Y strain. Furthermore, a posterior investigation studied the immunological effect of SLAMF1 deficiency in in vitro and in vivo models with different strains of different DTUs of *T. cruzi* [14]. All the strains, except VFRA (DTU VI), showed a decrease in parasite load in infected *Slamf1^-/-^* macrophages compared to BALB/c controls. Immunological studies in vivo by RT-qPCR with the Y strain displayed that, in the absence of SLAMF1, the immune response protected mice from the otherwise lethal Y infection. SLAMF1 deficiency favored a pro-inflammatory response involving CD4+ and CD8+ T cells, dendritic cells, and classically activated macrophages. However, in the case of the VFRA strain, no major changes were observed in the absence of SLAMF1. Thus, these results suggested that *T. cruzi* infection is SLAMF1-dependent, except for the VFRA strain. SLAMF1 would act during the infection of macrophages controlling parasite replication and therefore affecting survival in mice but in a strain-dependent manner.

Thus, to further characterize the role of SLAMF1, we performed a quantitative proteomic analysis to uncover the differences between BALB/c and *Slamf1^-/-^* macrophages in the in vitro infection by *T. cruzi*, identifying the different and common responses between Y and VFRA strains of this parasite.

## 2. Results

### 2.1. Quantitative Proteomics Analysis of BALB/c and Slamf1^-/-^ Macrophages Infected with the Y Strain of T. cruzi

We infected peritoneal BALB/c and *Slamf1^-/-^* macrophages for 24 h with two different strains, Y and VFRA, and we extracted the proteins of each condition for analysis by TMT-6plex. We detected a total of 1849 proteins for the quantitative study in all the conditions, and in the posterior analysis we established a threshold of <−0.5 and >0.5 for the log_2_ fold change and >1.3 for the −log_10_ (FDR). We chose 24 h post-infection (hpi) because this time has been established as the interval in which the parasite replicates inside the macrophage [14].

The Y strain was selected for three reasons: (1) the parasite load decreased in the *Slamf1^-/-^* macrophages; (2) the relevant changes in the immune response in these macrophages; (3) the SLAMF1-dependent lethality in infected susceptible BALB/c mice [14].

**Figure 1:**
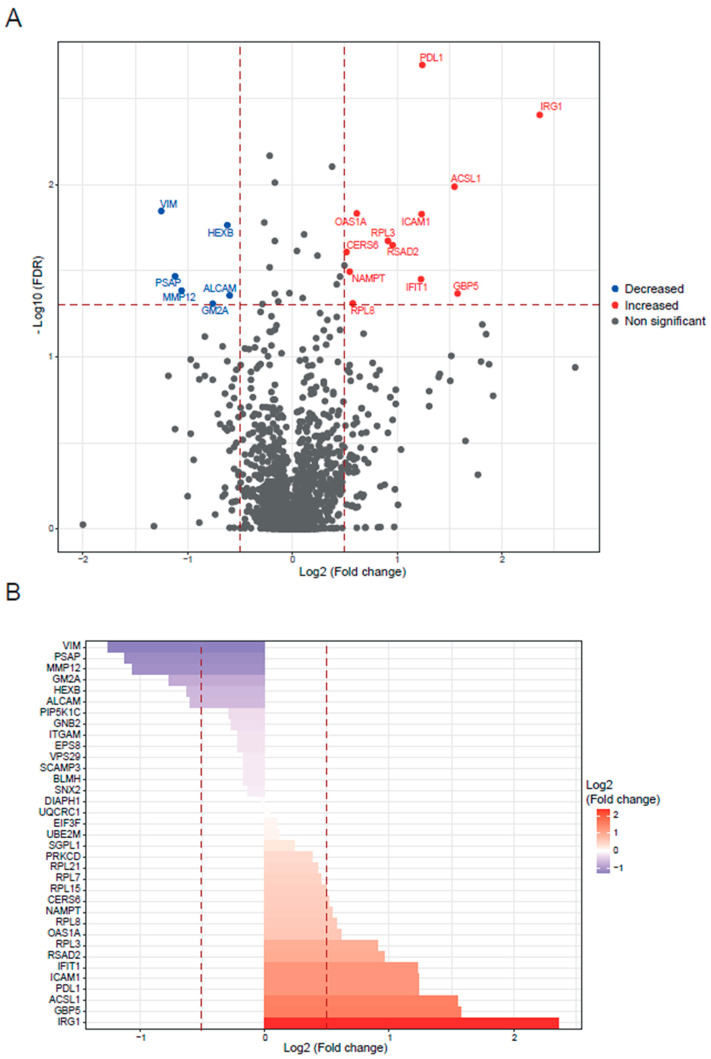
BALB/c macrophages infected by Y strain vs. non-infected BALB/c macrophages. (**A**) Volcano plot of detected proteins with log_2_ fold change in mean intensities and −log_10_ FDR of each protein. Thresholds of significance are shown as dashed lines: <−0.5 and >0.5 for the log_2_ fold change and >1.3 for the −log_10_ FDR. Significant down- or upregulated proteins are represented in blue or red, respectively. Non-significant proteins are marked in grey. (**B**) Bar plot of the significant proteins (>1.3 for the −log_10_ FDR) classified by log_2_ fold change. Thresholds are shown as dashed lines: <−0.5 and >0.5.

**Figure 2:**
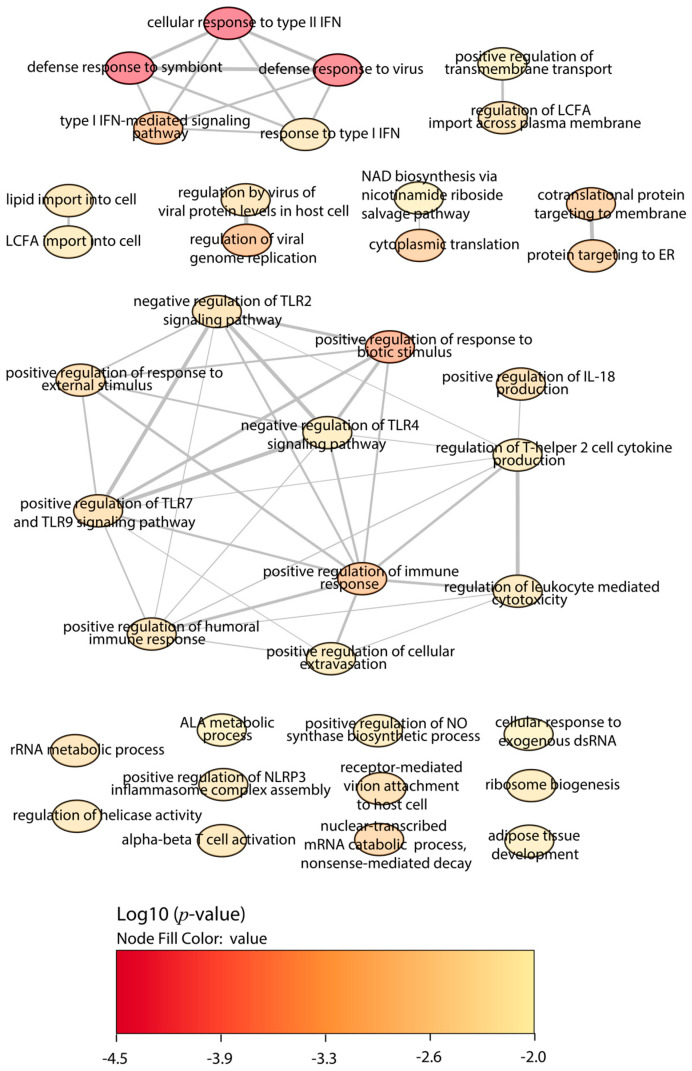
GO enrichment analysis by Enrichr webtool of upregulated proteins in BALB/c macrophages infected with the Y strain. Significant biological processes’ GO terms are displayed in bubbles. Highly similar GO terms are linked by edges, and the line width indicates the degree of similarity. Color is determined by the log_10_ (*p*-value), with red being the most and beige being the least statistically significant, respectively. LCFA, long-chain fatty acid; ALA, alpha-linolenic acid; TLR, toll-like receptor; IFN, interferon; IL, interleukin.

First, we focused on the BALB/c macrophages infected by the Y strain. After the quantitative analysis comparing with the control without infection, we obtained 18 significant proteins, 12 upregulated proteins, and 6 downregulated proteins (Figure 1). Then, we performed a gene ontology (GO) enrichment analysis to study the principal biological processes implicated in the Y strain infection with the significant proteins by fold change and FDR. We used the enrichment analysis tool from the Enrichr website and the “reduce and visualize gene ontology” (REVIGO) web tool to correlate the results. Considering the upregulated proteins (Figure 2), the most significant ontologies were related to the response against pathogens such as viruses and the IFN system (types I and II). Also, there was a slight negative regulation of TLR2 and TLR4 signaling pathways, while there was a positive regulation of TLR7 and TLR9 signaling pathways. Other significant GO terms were related to the lipid import, the protein translation pathways, the positive regulation of IL-18 production and the NACHT, LRR, and PYD domains-containing protein 3 (NLRP3) inflammasome complex assembly, the biosynthesis of nitric oxide synthase, and the alpha–beta T cell activation, among others.

**Figure 3:**
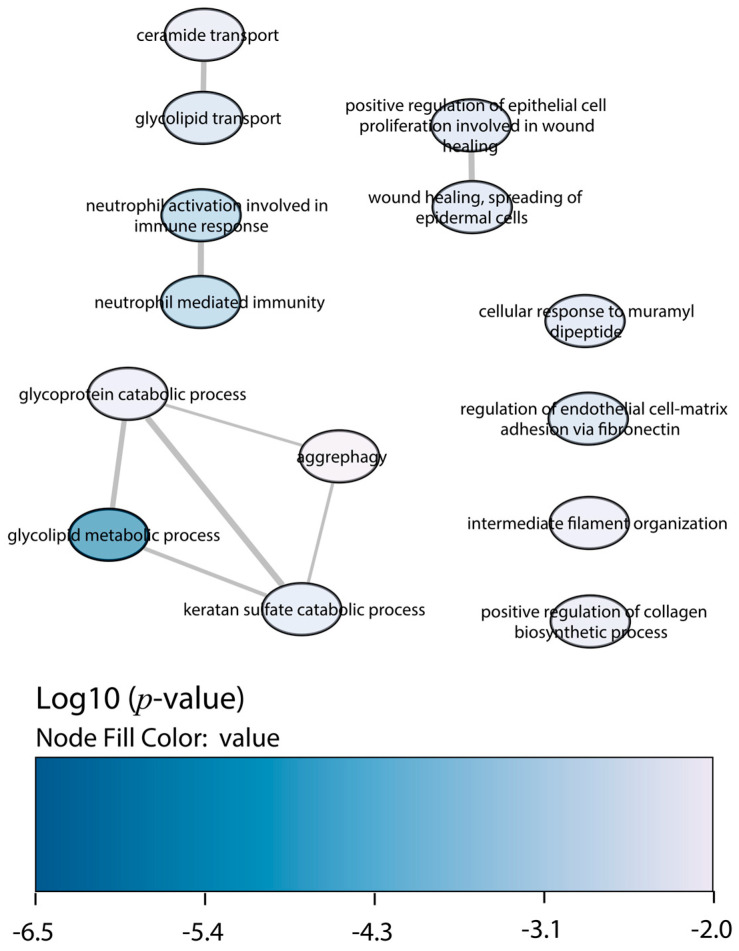
GO enrichment analysis by Enrichr webtool of downregulated proteins in BALB/c macrophages infected with the Y strain. Significant biological processes’ GO terms are displayed in bubbles. Highly similar GO terms are linked by edges, and the line width indicates the degree of similarity. Color is determined by the log_10_ (*p*-value), with blue being the most and white being the least statistically significant, respectively.

Regarding the downregulated proteins in BALB/c macrophages infected with the Y strain, the enrichment of biological processes displayed less significant GO terms than the upregulated proteins (Figure 3). The most relevant were the glycoprotein catabolic process, the glycolipid metabolic process, and the neutrophil-mediated immunity and activation.

**Figure 4:**
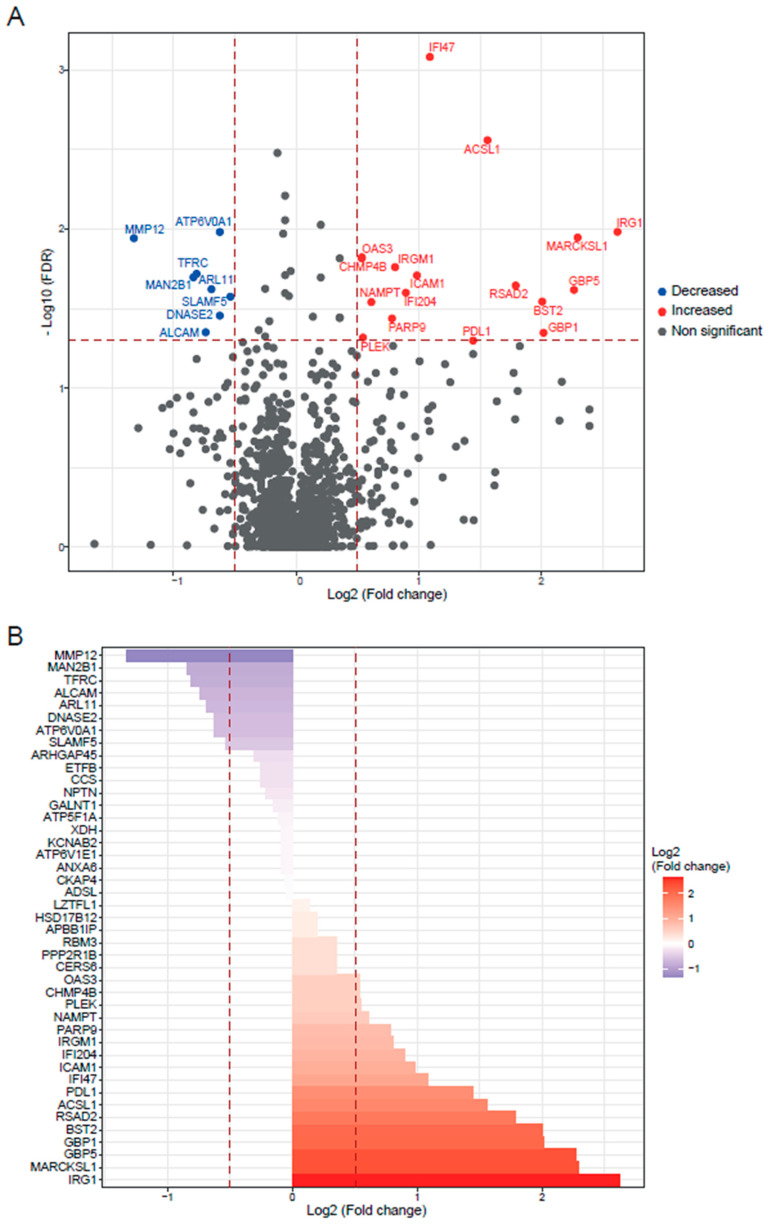
*Slamf1^-/-^* macrophages infected by Y strain vs. non-infected *Slamf1^-/-^* macrophages. (**A**) Volcano plot of detected proteins with log_2_ fold change in mean intensities and −log_10_ FDR of each protein. Thresholds of significance are shown as dashed lines: <−0.5 and >0.5 for the log_2_ fold change and >1.3 for the −log_10_ FDR. Significant down- or upregulated proteins are represented in blue or red, respectively. Non-significant proteins are marked in grey. (**B**) Bar plot of the significant proteins (>1.3 for the −log_10_ FDR) classified by log_2_ fold change. Thresholds are shown as dashed lines: <−0.5 and >0.5.

**Figure 5:**
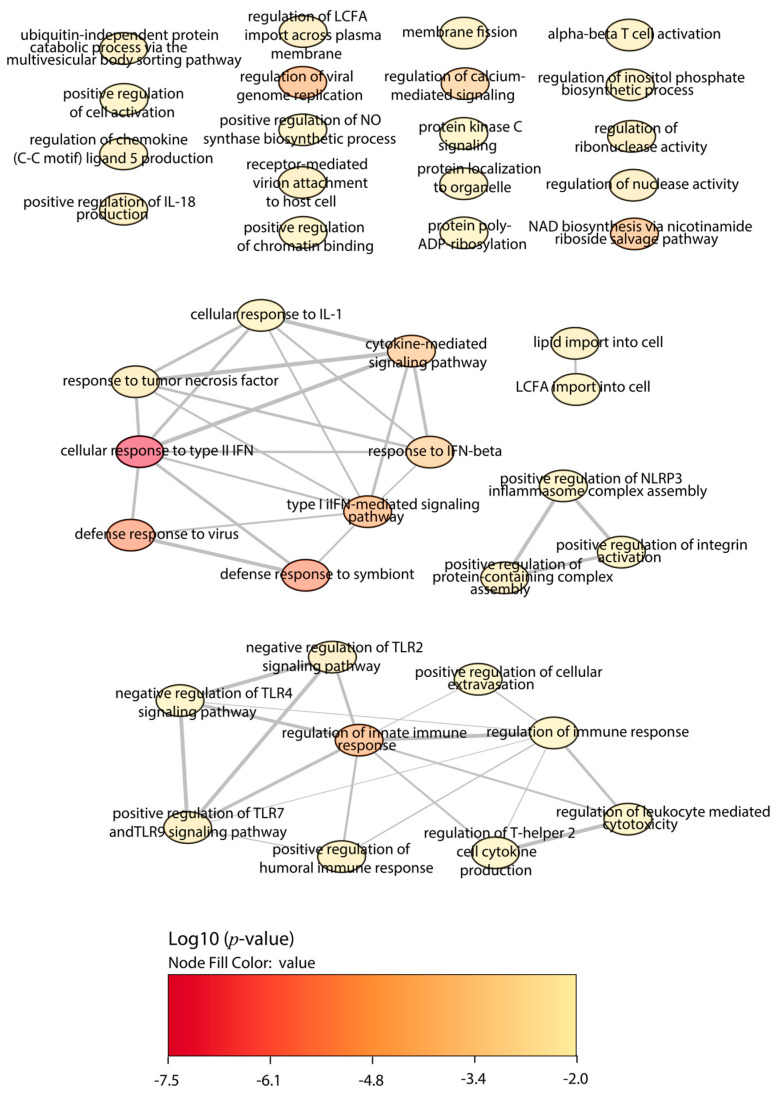
GO enrichment analysis by Enrichr webtool of upregulated proteins in *Slamf1^-/-^* macrophages infected with the Y strain. Significant biological processes’ GO terms are displayed in bubbles. Highly similar GO terms are linked by edges, and the line width indicates the degree of similarity. Color is determined by the log_10_ (*p*-value), with red being the most and beige being the least statistically significant, respectively. LCFA, long-chain fatty acid; ALA, alpha-linolenic acid; TLR, toll-like receptor; IFN, interferon; IL, interleukin.

The analysis of the *Slamf1^-/-^* macrophages infected with the Y strain revealed 25 significant proteins, 17 upregulated proteins, and 8 downregulated proteins (Figure 4). The GO enrichment analysis to study the principal biological processes implicated in the Y strain infection in the absence of the SLAMF1, considering the upregulated proteins (Figure 5), showed that the most significant GO terms were those related to the IFN types I and II, IL-1, TNF, IFN-β, NAD biosynthesis, cytokines signaling pathway, innate immune response, and the response against other pathogens as viruses. Also, other relevant GO terms were the alpha–beta T cell activation, the protein kinase C signaling, the negative regulation of TLR2 and TLR4, and the positive regulation of TLR7, TLR9, NLRP3 inflammasome complex assembly, nitric oxide synthase, and IL-18.

**Figure 6:**
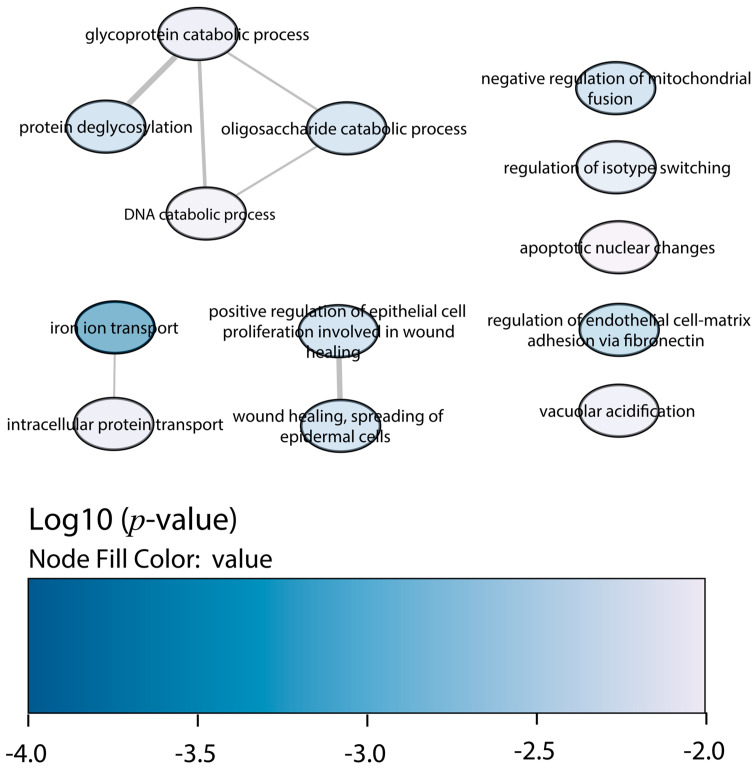
GO enrichment analysis by Enrichr webtool of downregulated proteins in *Slamf1^-/-^* macrophages infected with the Y strain. Significant biological processes’ GO terms are displayed in bubbles. Highly similar GO terms are linked by edges, and the line width indicates the degree of similarity. Color is determined by the log_10_ (*p*-value), with blue being the most and white being the least statistically significant, respectively.

Regarding the downregulated proteins in *Slamf1^-/-^* macrophages infected with the Y strain, the GO enrichment also displayed less significant ontologies than the upregulated proteins (Figure 6). The most relevant GO terms were the iron ion transport, the negative regulation of mitochondrial fusion, the protein deglycosylation, and the glycoprotein and oligosaccharide (OS) catabolic processes, among others.

### 2.2. Quantitative Proteomics Analysis of BALB/c and Slamf1^-/-^ Macrophages Infected with the VFRA Strain of T. cruzi

The VFRA was selected since it infects the macrophages independent of SLAMF1 and exhibits an opposite behavior than the Y strain. While Y strain parasite loads decrease in the *Slamf1^-/-^* macrophages, the VFRA strain parasite loads remain unchanged. Furthermore, VFRA produces a chronic infection in mice, and the immune response associated with the VFRA infection is different from the Y strain response [14].

We performed the same analysis with the macrophages infected with the VFRA strain of *T. cruzi*. We obtained 12 significant proteins in the BALB/c infected macrophages: 6 upregulated and 6 downregulated (Figure S1); in the *Slamf1^-/-^* macrophages 19 significant proteins, 9 upregulated and 10 downregulated (Figure S2).

Regarding the GO enrichment with the upregulated proteins in the BALB/c macrophages infected with the VFRA strain, the most significant GO terms were the following: IFN type I and II responses, cellular response to IFN-β and IL-1, negative regulation of TLR2 and TLR4, autophagy of peroxisome and pexophagy, lipid import, response to mitochondrial depolarization and positive regulation of NLRP3 inflammasome complex assembly, and IL-18 and transmembrane transport, among others (Figure S3).

Considering the downregulated proteins in BALB/c macrophages infected with the VFRA strain, the enrichment of biological processes showed that the most relevant were glycoprotein, glycosphingolipid (GSL), and OS catabolic processes, negative regulation of mitochondrial fusion, protein deglycosylation, and neutrophil activation (Figure S4).

In the GO enrichment analysis in the *Slamf1^-/-^* macrophages infected with the VFRA strain, with the upregulated proteins, the most significant GO terms were the following: IFN type I and II responses, NAD biosynthesis, lipid import, the negative regulation of TLR2 and TLR4, and the positive regulation of NLRP3 inflammasome complex assembly, nitric oxide synthase, and IL-18 (Figure S5).

Finally, considering the downregulated proteins in *Slamf1^-/-^* macrophages infected with the VFRA strain, the enrichment of biological processes showed that the most relevant GO terms were the protein deglycosylation and the glycoprotein, GSL, and OS catabolic processes, among others (Figure S6).

### 2.3. Comparison between the Experimental Conditions and Protein–Protein Interaction Networks

**Figure 7:**
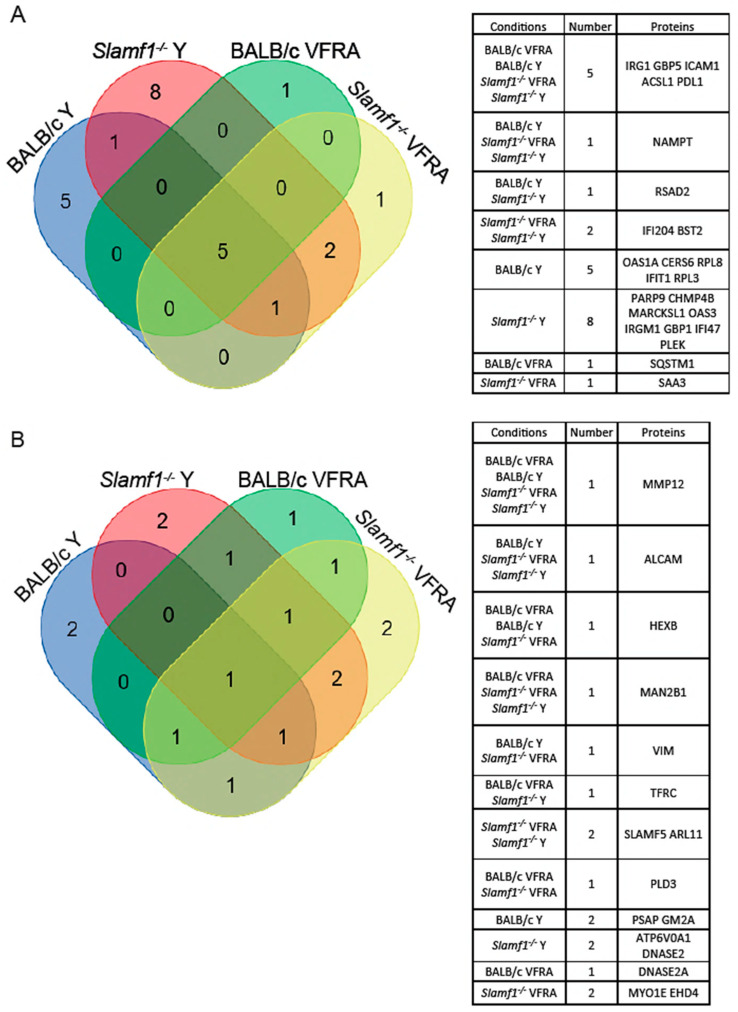
Venn diagrams of shared proteins between macrophage conditions. (**A**) Upregulated proteins. (**B**) Downregulated proteins. Tables display the shared proteins in each combination.

To study the common and specific traits between the conditions with significant quantified proteins, we represented the data in a Venn diagram (Figure 7). Five upregulated proteins were common to all the conditions: immune-response gene 1 (IRG1), guanylate-binding protein (GBP) 5, intercellular adhesion molecule 1 (ICAM1), acyl-CoA synthetase long-chain family member 1 (ACSL1), and programmed cell death protein ligand 1 (PDL1); and one downregulated: metalloproteinase 12 (MMP12). Independently of SLAMF1, with the Y strain infection, there was one common upregulated protein, the radical S-adenosyl methionine domain containing 2 (RSAD2), and with the VFRA strain there was one common downregulated protein, the phospholipase D3 (PLD3). Moreover, independently of the strain, with the *Slamf1^-/-^* macrophages there were two common upregulated proteins, interferon-activable protein 204 (IFI204) and bone marrow stromal antigen 2 (BST2), and two common downregulated proteins, SLAMF5 and ADP ribosylation factor-like GTPase 11 (ARL11). Interestingly, *Slamf1^-/-^* macrophages infected by the Y strain displayed 10 unique significant proteins, 8 of them upregulated, while BALB/c macrophages infected by the Y strain had 7 unique proteins, and the other 2 conditions (*Slamf1^-/-^* and BALB/c macrophages infected by VFRA strain) 3 and 2, respectively.

Then, STRING was used to study the relationship between the distinct proteins detected in our experimental conditions (Figure S7). The analysis showed more relationships in upregulated proteins than in the downregulated ones. Our results displayed some proteins (IRG1, GBP5, PDL1, and ICAM1) correlated in all the conditions forming a unique section, in combination with other proteins in each specific condition, such as RSAD2, presented only in the Y infections.

Also, the association of SLAMF5 and MMP12 in *Slamf1^-/-^* macrophages was remarkable, whereas MMP12 was alone in the BALB/c macrophages, and ICAM1 was associated with ALCAM, an upregulated protein, and/or TFRC, a downregulated protein.

Furthermore, we represented in Venn diagrams the upregulated and downregulated enriched GO terms of each condition. Our objective was to see which biological processes were common or unique considering the absence of SLAMF1 and the two different strains of *T. cruzi*.

**Figure 8:**
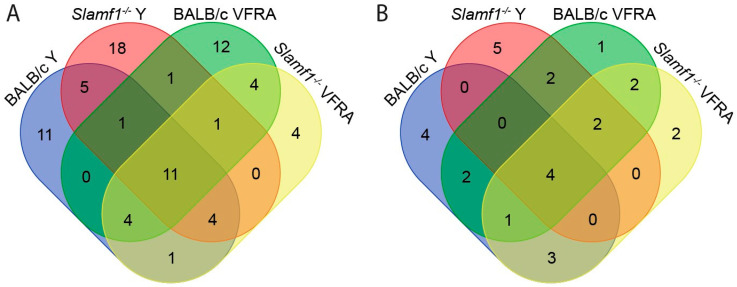
Venn diagrams of enriched GO terms shared between macrophage conditions. (**A**) Enriched upregulated GO terms. (**B**) Enriched downregulated GO terms.

Considering the upregulated GO terms (Figure 8A and Table S1), all the conditions shared 11 GO terms, such as the negative regulation of TLR2 and TLR4 signaling pathways and the positive regulation of IL-18 production and NLRP3 inflammasome complex assembly. Remarkably, all the conditions displayed different GO terms related to IFN types I and II. Although the distinct GO terms are slightly different from each other, the activation of the IFN system could be added as a shared enriched pathway in all the infections in this study.

Regarding the Y strain infections, independently of the presence/absence of SLAMF1, there are five GO terms shared, like the positive regulation of TLR7 and TLR9 signaling pathways and the alpha–beta T cell activation. Interestingly, both *Slamf1^-/-^* macrophages Y and VFRA infections and BALB/c macrophages infected by VFRA shared the response to IFN-β, suggesting that in the BALB/c with the Y strain, this protective immune response is not so effective, as in VFRA the absence of SLAMF1 would allow the correct activation of this pathway.

Notably, there was a great quantity of unique upregulated GO terms for each condition (Table S2). BALB/c macrophages infected by the Y strain displayed 11 unique GO terms, *Slamf1^-/-^* macrophages infected by the Y strain 18 GO terms, BALB/c macrophages infected by the VFRA strain 12 GO terms, and *Slamf1^-/-^* macrophages infected by VFRA strain only 4 GO terms. In BALB/c macrophages infected by the Y strain, we found many unique GO terms related to the transcription–translation processes of the cell. However, in the other conditions, the unique GO terms were varied, and it was impossible to establish patrons or specific profiles. Only in the BALB/c infected by VFRA, 3 out of 12 GO terms were related to autophagy, suggesting that this process could be enriched in a normal VFRA infection.

The Venn diagram with the downregulated GO terms is shown in Figure 8B, and Table S3 lists the shared enriched GO terms of this analysis. All the conditions shared four enriched GO terms, although two were related to wound healing, not Chagas disease. However, all shared the glycoprotein catabolic process, suggesting that *T. cruzi* may inhibit glycoprotein degradation in the cell host. All the conditions except BALB/c infected by the Y strain displayed downregulation of the protein deglycosylation and OS catabolism. Interestingly, only in the BALB/c infections, independently of the strain, did we find markers of activation of neutrophils. This means that the neutrophil’s role in the innate immune response against *T. cruzi* is downregulated in a normal infection, but the depletion of SLAMF1 avoids it. Also, in the VFRA infections, independently on SLAMF1, GSL catabolism was detected, which would be a specific molecular process decreased only by this strain.

Finally, Table S4 compiles unique enriched downregulated GO terms in each condition. In BALB/c macrophages infected with the Y strain, there were four unique GO terms: ceramide transport, glycolipid transport, metabolism, and aggrephagy. Although this condition was the only one without deglycosylation or OS catabolism, the specific response of Y infection seems to be more correlated with the glycolipids and ceramides, two important molecules of the cell–host membrane. Also, this infection specifically produces the process known as aggrephagy, a type of macroautophagy of protein aggregates. Among the other unique GO terms, *Slamf1^-/-^* macrophages infected by the Y strain were the most relevant. It presented five unique GO terms, without relation between them. It displayed downregulation of iron transport, intracellular protein transport, the catabolism of DNA, nuclear apoptosis, and vacuolar acidification.

### 2.4. Functional Enrichment Analysis

**Figure 9:**
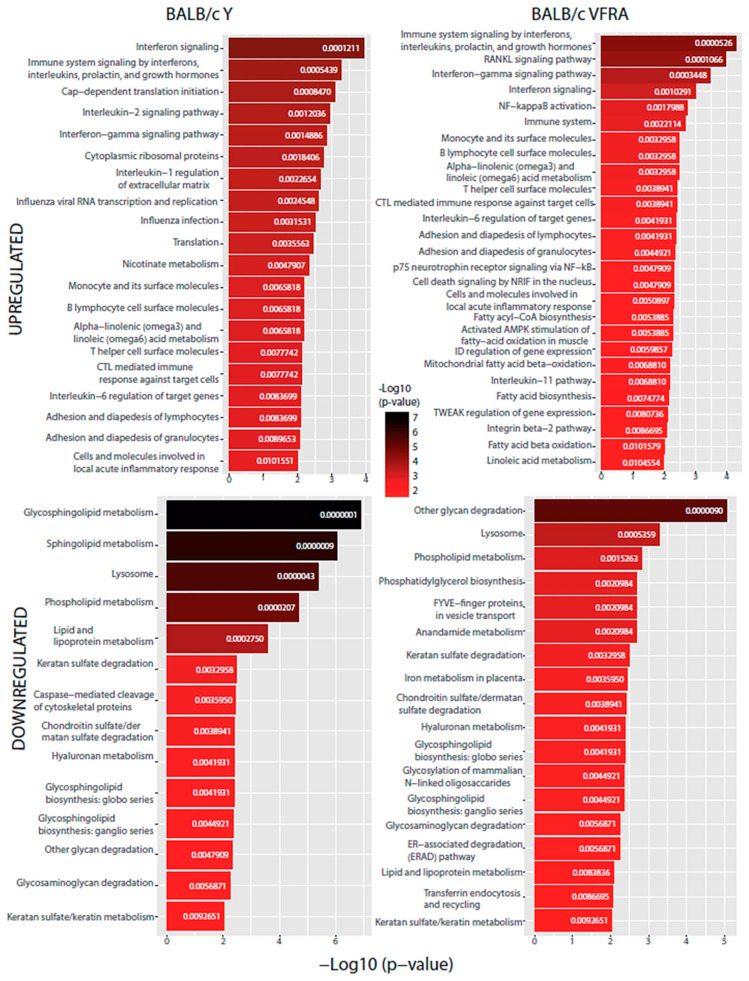
BioPlanet database functional enrichment analysis of BALB/c macrophages. Significant pathways of BALB/c macrophages infected by the Y strain and the VFRA strain according to the significantly upregulated and downregulated proteins.

**Figure 10:**
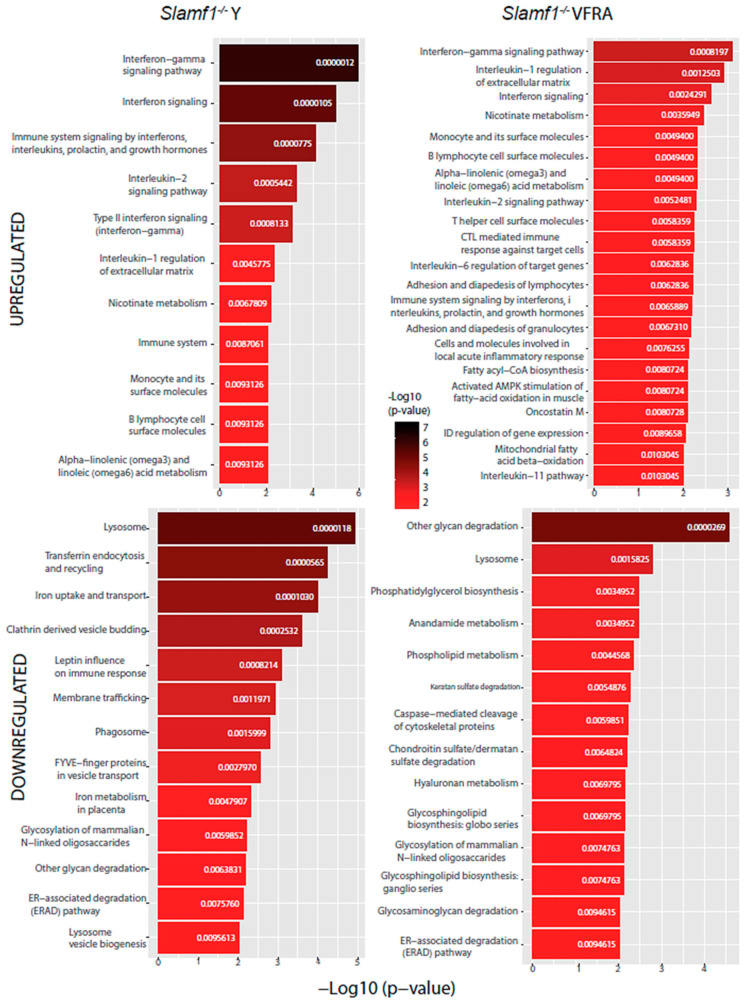
BioPlanet database functional enrichment analysis of *Slamf1^-/-^* macrophages. Significant pathways of *Slamf1^-/-^* macrophages infected by the Y strain and the VFRA strain according to the significantly upregulated and downregulated proteins.

Our last analysis aimed to predict which specific cellular pathways could be affected by the proteins with significant changes. We performed a functional enrichment analysis with the Enrichr web tool using the BioPlanet database (Figure 9 and Figure 10).

All the conditions shared the IFN signaling pathway, as we had already seen in the enrichment GO analysis, confirming that, as in virus infections, *T. cruzi* activates IFN signaling in the host. Specifically, the IFN-γ pathway was increased in all of them in this analysis. Also, we found in every condition, except in the *Slamf1^-/-^* macrophages infected by the Y strain, the upregulation of IL-6, while in all of them except the BALB/c macrophages infected by the VFRA strain, we had upregulated the IL-1β regulation of the extracellular matrix and IL-2 signaling pathway. Furthermore, in all the VFRA infections, independently of SLAMF1 expression, we had an upregulation of the IL-11 pathway. In BALB/c macrophages infected by the Y strain, we obtained pathways related to the translation process and the replication and infection of viruses, among others, while in BALB/c macrophages infected by the VFRA strain we found mostly pathways related to the nuclear factor kappa-light-chain-enhancer of activated B cells (NF-κB) activation.

Regarding the downregulated enriched pathways, all the conditions shared other glycan degradation and lysosome pathways. Also, we found in every condition, except in the *Slamf1^-/-^* macrophages infected by the Y strain, the downregulation of GSL biosynthesis and glycosaminoglycan degradation, while in all of them, except the BALB/c macrophages infected by the Y strain, we had the downregulation of the endoplasmic-reticulum-associated degradation pathway. Furthermore, in all the VFRA infections, independently of SLAMF1 expression, there was a downregulation of phosphatidylglycerol (PG) biosynthesis. Finally, specifically in the *Slamf1^-/-^* macrophages infected by the Y strain, we obtained a downregulation of pathways related to membrane trafficking, clathrin vesicles, iron uptake, lysosome biogenesis, or phagosome.

## 3. Discussion

Previously to this work, a decreased parasite replication in *Slamf1^-/-^* macrophages infected with Dm28, Y, 10R26, M6421, and Bug2148 strains was detected compared to BALB/c macrophages at 24 hpi, indicating that SLAMF1 is necessary for the internalization and intracellular replication of these strains [14]. However, VFRA displayed the opposite behavior. Thus, we performed a proteomic study on the effect of the absence of SLAMF1 in macrophages after Y and VFRA strains of in vitro infection.

We detected some significantly upregulated proteins upon infection: IRG1, GBP5, ICAM1, ACSL1, and PDL1 independently on the presence or absence of SLAMF1. IRG1 is a novel M1 macrophage marker and produces itaconic acid that inhibits the isocitrate lyase, the key enzyme of the glyoxylate shunt [16], necessary in *T. cruzi* to survive on limited carbon conditions [17]. Hearts of BALB/c mice infected with the Y strain displayed high levels of itaconic acid production [18], and increased levels of *Irg1* were detected in the heart tissue and intestine of Y and VFRA-infected BALB/c and *Slamf1^-/-^* mice [14]. Thus, our results support further research into itaconic acid and its therapeutic potential for treating *T. cruzi* infection.

GBP5 and other GBPs are strongly induced by IFN-γ, a cytokine increased in cells and organs infected by *T. cruzi* [14,19,20]. GBPs facilitate cell-autonomous immunity through inflammasome activation and/or microbe elimination [21,22]. We have described for the first time the relationship between GBP5 and this parasite. *T. cruzi* infections also displayed upregulation of ICAM1 (CD54) [23,24], which deficiency generates a higher infection susceptibility and a decrease in T cell recruitment [25]. Our results support the role of ICAM1 as a defense mechanism against *T. cruzi*. On the other hand, our study is the first that correlates the increase in ACSL1 with Chagas disease. Interestingly, ACSL1 increases in acute myocardial infarction patients [26], which is one of the symptoms of chronic Chagas disease.

The PD1/PDL1 pathway inhibits the proliferation and activation of macrophages leading to T cell anergy [27], while its blockade reinvigorates exhausted CD8+ T cells, reducing the pathogen burden [28,29]. However, there are some controversies considering its role in *T. cruzi* infections. Some researchers have shown that PD1 inhibition leads to reduced parasitemia [30], but others have described that PDL1 inhibition favors a higher parasitemia [31]. On the other hand, PD1–PDL1 interaction protected against heart damage in chronic *T. cruzi* infection, reducing the exacerbated immune response without modifying the parasite load [32], and increased PDL1 levels were detected in BALB/c macrophages as in our proteomic study [33]. Altogether, the PD1/PDL1 pathway may regulate the activation of infected macrophages to prevent immune system exacerbation.

Regarding the downregulated protein found in all infections, MMP12, an increase was detected in *T. cruzi* infections at 24 hpi in mouse macrophages [34], contrary to our proteomic analysis. However, the same report analyzed the expression at 48 hpi too, revealing a significant reduction in MMP12. These different results could be due to the different parasite-infecting strains used. Moreover, MMP12 was identified as a resolution-promoting factor in myocardial infarction, a symptom of chronic Chagas disease, inducing M2 macrophages and the secretion of anti-inflammatory cytokines [35]. Thus, the decreased expression of MMP12 would enhance the cardiac disease preventing inflammation resolution.

Considering the strain-specific proteins without a dependency on SLAMF1, Y strain infections displayed one common upregulated protein (RSAD2), and VFRA strain infections had one common downregulated protein (PLD3). RSAD2 is an IFN-stimulated protein that restricts various families of viruses [36,37], while PLD3 digests ssRNA and ssDNA in lysosomes/endosomes in viral and bacterial infections [38,39]. This is the first time both proteins have been related to *T. cruzi* infections.

Addressing the effect of SLAMF1 using the *Slamf1^-/-^* macrophages, we detected two common upregulated proteins (IFI204 and BST2) and two common downregulated proteins (SLAMF5 and ARL11), independently on the infecting strain. IFI204 is orthologous to the human IFN-γ inducible protein 16 (IFI16), which was increased in infected dendritic cells [40], while BST2 is induced by type I IFN and traps viral particles onto the cell surface [41]. Our study points to IFI204/IFI16 and BST2 as potential Chagas disease targets and SLAMF1 as their inhibitor through some unknown mechanism. On the other hand, SLAMF5 produces IL-6 and TNF-α [42], while ARL11 regulates pro-inflammatory effectors [43] and is associated with M2 macrophages [44]. Lower *Tnf* levels were described in *Slamf1^-/-^* macrophages infected with Y and VFRA and the switch to M1 phenotype [14]. Decreased levels of both SLAMF5 and ARL11 agree with those results, although more studies must be conducted to unravel the mechanisms that connect SLAMF1 to them.

*Slamf1^-/-^* macrophages infected by the lethal Y strain displayed the biggest number of altered unique proteins. These proteins either blocked or induced by SLAMF1 may be involved in the disappearance of this strain’s lethality in the absence of SLAMF1, making them attractive molecules to understand and avoid pathogenicity. In contrast, *Slamf1^-/-^* macrophages infected by the VFRA strain only displayed three significant unique proteins. This agrees with previous observations that VFRA barely changes the cytokine production in *Slamf1^-/-^* macrophages [14].

Among these unique significant proteins in *Slamf1^-/-^* macrophages infected by the Y strain, several have been described as macrophage response regulators: PARP9, MARCKSL1, IRGM1, ATP6V0A1, GBP1, and IFI47 [45,46,47,48,49,50,51]. They would contribute to the innate immune response promoting macrophage activation and the killing of the parasite. This agrees with the reduced parasite load in *Slamf1^-/-^* macrophages infected by the Y strain [14], suggesting that SLAMF1 signaling after the *T. cruzi* interaction affects these critical proteins to resolve the infection. Alternatively, the Y strain may promote changes in them to persist in the cell, but the decrease in its internalization by the absence of SLAMF1 reduces the effect. This points out the relevance of SLAMF1 and these proteins as possible therapeutic targets.

To further understand the relevance of our results, we performed GO and functional enrichment analysis. Table 1 summarizes the most relevant macrophage pathways suffering a positive or negative regulation. We detected nine common pathways in all the conditions without an SLAMF1 effect, some implicated in innate immunity. IFN responses activate parasite killing through microbicidal molecules and ROS in the early stages of *T. cruzi* infection [52]. Also, NLRP3 inflammasome induces the secretion of the inflammatory cytokines IL-1β and IL-18, which guide pyroptosis and nitric oxide production, favoring *T. cruzi* elimination [53]. Our study supports the key role of these processes in dealing with the *T. cruzi* infection in macrophages independently of the strain.

Also, we found an increase in the alpha-linolenic acid (ALA) and linoleic acid (LA) metabolism. LA is a precursor of omega-6 fatty acids with predominantly pro-inflammatory actions, while ALA is a precursor of omega-3 fatty acids with mostly anti-inflammatory effects [54]. Their increment may be due to the homeostatic mechanisms of inflammation. Other changes in lipids included downregulation of the GSL catabolism and PG biosynthesis in VFRA-infected macrophages. GSLs are critical for the immune response and the parasite phagocytosis [55] while PG displays anti-inflammatory effects [56]. Thus, these downregulations would prevent parasite proliferation in macrophages and contribute to the persistence of the pro-inflammatory response against the VFRA strain.

Finally, other responses were also specific to parasite strain or SLAMF1-dependent strain. IL-11 activation was VFRA-specific, being upregulated in infections by Tulahuen strain [57], which belongs to the DTU VI as VFRA, suggesting that IL-11 activation would be DTU-specific. TLR7 and TLR9 signaling was specifically increased with Y strain, while the downregulation of the TLR2 and TLR4 signaling was found in all the conditions. TLR2 and TLR4 sense molecules of the trypomastigote surface inducing the nitric oxide and pro-inflammatory cytokine production [52], and their downregulation could be negative feedback signaling to avoid an over-enhanced immune response or a regulation mechanism induced by the parasite. On the other hand, TLR9 induces cytokine production and activation of Th1 responses [52], and a triple defect in TLR3, 7, and 9 made mice more susceptible to the infection [58]. Thus, we have pointed out that the Y strain, which causes mice mortality, enhances TLR signaling, while VFRA, a non-lethal strain in mice, does not produce this effect in macrophages. Moreover, as TLR9 recognized CpG DNA and TLR7 ssRNA in the lysosomes, this suggests a bigger exposure to parasite intracellular content due to better *T. cruzi* degradation. This would correlate with a previous proteomic study in which VFRA displayed more antioxidant molecules than the Y strain [59]. As VFRA is more resistant to these killing processes, it would release less DNA and ssRNA in the lysosome, explaining the increase in TLR7 and TLR9 activation only with the Y strain infection.

In summary, we have identified different down- or upregulated proteins in Y and VFRA infections in macrophages. Some are SLAMF1-dependent and/or strain-dependent, whereas the others are common to all *T. cruzi* infections. We have highlighted the relevance of most of them as critical innate immune regulators and displayed the significant functional pathways in which they participate, opening new avenues of research in Chagas disease. Independently on SLAMF1, *T. cruzi* induces different responses in macrophages to deal with the infection and kill the parasite, such as the type I and II IFN responses, NLRP3 inflammasome activation, IL-18 production, TLR7 and TLR9 activation specifically in the Y strain infection, and IL-11 signaling specifically in the VFRA strain infection.

## 4. Materials and Methods

### 4.1. Parasite Culture

*T. cruzi* VFRA strain was obtained from Dr. M. Miles (London School of Hygiene and Tropical Medicine, London, UK) through the European program ChagasEpiNet. Y strain was obtained from Harvard Medical School (Boston, MA, USA). This strain was isolated originally from a human patient in Brazil [60].

Trypomastigotes of *T. cruzi* were co-cultured in vitro with Vero cells (ATCC number CCL-81). Vero cells were cultured with complete Roswell Park Memorial Institute (RPMI, Thermo Fisher Scientific, Waltham, MA USA) medium containing 2 mM L-glutamine, 100 U/mL penicillin, 1000 U/mL gentamicin, 10 μg/mL streptomycin, and 0.1 mM non-essential amino acids and supplemented with 5% fetal bovine serum (FBS, Gibco Life Technologies, Grand Island, NY, USA). Cells were maintained at 37 °C in an atmosphere of 5% CO_2_ until the cells reached 80% confluence in the biosafety level 3 (BSL3) cell culture laboratories of the Centro de Biología Molecular Severo Ochoa (Madrid, Spain). Then, Vero cells were infected with trypomastigotes following a 10:1 parasite–cell ratio. After 4 days, the supernatant of the infected cells with the trypomastigotes was collected, dead cells and amastigotes were removed by centrifugation at 1000× *g* for 5 min, and trypomastigotes were collected by centrifugation at 1600× *g* for 10 min. These trypomastigotes were used for macrophage infections.

### 4.2. Mice and Ethic Statement

Female mice of 10–12 weeks old were used. *Slamf1^-/-^* mice were obtained from Dr. Cox Terhost (Harvard Medical School, Boston, MA, USA), while BALB/c mice were obtained from Charles River Laboratories. Mice were maintained under pathogen-free conditions at *Centro de Biología Molecular Severo Ochoa* (Madrid, Spain) animal facility.

This study was carried out in strict accordance with the European Commission’s legislation for the protection of animal use purposes (2010/63/EU). The protocol for the treatment of the animals was approved by the “*Comité de Etica de Investigación de la Comunidad de Madrid*” (Spain, permits PROEX 148/15, and PROEX 213.6/21). Animals had unlimited access to food and water. They were euthanized in a CO_2_ chamber and all efforts were made to minimize their suffering.

### 4.3. Isolation of Peritoneal Macrophages and In Vitro Infection

BALB/c and *Slamf1^-/-^* 10–12 weeks-old female mice were injected intraperitoneally with 10% thioglycollate (Gibco, Grand Island, NY, USA) (1 mL per mouse). After 4 days, mice were euthanized by CO_2_ inhalation. Peritoneal cells were collected by lavage with 10 mL of cold phosphate-buffered saline (PBS), and they were pelleted by centrifugation (260× *g*, for 10 min, at 4 °C). Then, they were seeded in p100 plates (10^7^ cells/plate) in RPMI medium supplemented with 2 mM L-glutamine, 100 U/mL penicillin, 1000 U/mL gentamicin, 10 μg/mL streptomycin, 0.1 mM non-essential amino acids, and 5% FBS overnight at 37 °C in an atmosphere of 5% CO_2_. Non-adherent cells were removed by gently washing three times with warm PBS. These peritoneal macrophages were infected with trypomastigotes of Y or VFRA strains at a ratio of 10 parasites per cell in the BSL3 cell culture laboratories of the Centro de Biología Molecular Severo Ochoa (Madrid, Spain). At 24 hpi, supernatant with the trypomastigotes was eliminated and the macrophages, including the control macrophages without infection, were washed three times with PBS to remove the unbound parasites.

### 4.4. Protein Extraction and Digestion for the Proteomic Analysis

For protein extraction, three cell replicates (*n* = 3) for each condition were incubated in RIPA buffer (150 mM NaCl, 20 mM Tris, pH = 7.6, 0.5% sodium deoxycholate, 1 mM EDTA, 0.1% SDS, 1% Triton-X100) with protease (cOmplete tablets Easypack, Roche, Basel, Switzerland) and phosphatase (PhosSTOP, Roche, Basel, Switzerland) inhibitors for 30 min at 4 °C and centrifuged at 20,800× *g* for 15 min, and the supernatants were kept. For protein quantification, bicinchoninic acid assay (BCA Protein Assay, Thermo Fisher Scientific, Waltham, MA USA) was utilized using supernatants, and extrapolation was based on a standard curve of known concentrations of bovine serum albumin.

Protein extracts were prepared in up to 50 µL of sample reduction buffer and then applied onto 1.2 cm wide wells of a conventional SDS-PAGE gel (0.75 mm thick, 4% stacking, and 10% resolving). The run was stopped when the front entered 3 mm into the resolving gel so that the whole proteome became concentrated in the stacking/resolving gel interface. The unseparated protein bands were visualized by Coomassie staining, excised, cut into cubes (2 × 2 mm), and placed in 0.5 mL microcentrifuge tubes [61]. The gel pieces were destained in acetonitrile/water (1:1), were reduced and alkylated (disulfide bonds from cysteinyl residues were reduced with 10 mM DTT for 1 h at 56 °C, and then thiol groups were alkylated with 10 mM iodoacetamide for 30 min at room temperature in darkness), and digested in situ with sequencing grade trypsin (Promega, Madison, WI, USA) as described by Shevchenko et al. [62], with minor modifications. The gel pieces were shrunk by removing all liquid using sufficient acetonitrile. Acetonitrile was pipetted out, and the gel pieces were dried in a speed vac. The dried gel pieces were re-swollen in 100 mM Tris-HCl pH 8, 10 mM CaCl_2_ with 60 ng/µL trypsin at 5:1 protein/enzyme (*w*/*w*) ratio. Digestion was performed in the presence of 0.2% RapiGest (Waters, Milford, MA, USA). The tubes were kept on ice for 2 h and incubated at 37 °C for 12 h. Digestion was stopped by the addition of 1% trifluoroacetic acid. Whole supernatants were dried down and then desalted onto OMIX Pipette tips C18 (Agilent Technologies, Santa Clara, CA, USA) until the mass spectrometric analysis was performed.

### 4.5. TMT Labeling and Fractionation

The resultant peptide mixture from desalted proteins tryptic digest (50 µg) was labeled using chemicals from the TMT sixplex Isobaric Mass Tagging Kit (Thermo Fisher Scientific, Waltham, MA, USA) as described by the manufacturer. Briefly, peptides were dissolved in 50 μL of 100 mM triethylammonium bicarbonate, adjusted to pH 8. For labeling, each TMT reagent was dissolved in 41 μL of acetonitrile and added to the respective peptide mixture and then incubated at room temperature for one hour. Labeling was stopped by the addition of 8 μL 5% hydroxylamine. Whole supernatants were dried down, and the six samples were mixed to obtain the “6plex-labeled mixture”. The mixture was analyzed by Reverse-Phase Liquid Chromatography–Tandem Mass Spectroscopy (RP-LC-MS/MS) to check the efficiency of the labeling.

The sample was then fractionated using the Pierce High pH Reversed-Phase Peptide Fractionation Kit (Thermo Fisher Scientific, Waltham, MA, USA) as described, with minor modifications. The sample was re-swollen in 0.1% trifluoroacetic acid and then loaded onto an equilibrated, high-pH, reversed-phase fractionation spin column. A step gradient of increasing acetonitrile concentrations (5–50%) in a volatile high pH (Triethylamine (0.1%)) was then applied to the columns to elute bound peptides into nine different fractions collected by centrifugation. The fractions obtained from a high-pH, reversed-phase 6plex-labeled mixture were dried and stored until analysis by mass spectrometry for quantification.

### 4.6. Analysis by RP-LC-MS/MS

The fractions were resuspended in 10 µL of 0.1% FA and analyzed by RP-LC-MS/MS in an Easy-nLC II system coupled to an ion trap LTQ-Orbitrap-Velos-Pro hybrid mass spectrometer (Thermo Scientific, Waltham, MA, USA). The peptides were concentrated by reverse-phase chromatography using a 0.1 mm × 20 mm C18 RP precolumn (Thermo Fisher Scientific, Waltham, MA, USA) and then separated using a 0.075 mm × 250 mm bioZen 2.6 µm Peptide XB-C18 RP column (Phenomenex, Alcobendas, Spain) operating at 0.25 μL/min. Peptides were eluted using a 90 min dual gradient. The gradient profile was set as follows: 5–25% solvent B for 68 min, 25–40% solvent B for 22 min, 40–100% solvent B for 2 min, and 100% solvent B for 18 min (solvent A: 0.1% FA in water; solvent B: 0.1% FA, 80% acetonitrile in water). ESI ionization was performed using a Nano-bore emitters Stainless Steel ID 30 μm (Proxeon, Waltam MA, USA) interface at 2.1 kV spray voltage with S-Lens of 60% [63].

The instrument method consisted of a data-dependent top-20 experiment with an Orbitrap MS1 scan at a resolution (m/Δm) of 30,000 followed by twenty higher-energy collision dissociation (HCD) MS/MS mass-analyzed in the Orbitrap at 7500 (Δm/m) resolution. MS2 experiments were performed using HCD to generate high-resolution and high-mass-accuracy MS2 spectra. The minimum MS signal for triggering MS/MS was set to 500. The lock mass option was enabled for both MS and MS/MS mode, and the polydimethylcyclosiloxane ions (protonated (Si(CH_3_)_2_O))_6_; *m*/*z* 445.120025) were used for internal recalibration of the mass spectra.

Peptides were detected in survey scans from 400 to 1600 amu (1 μscan) using an isolation width of 1.3 u (in mass-to-charge ratio units), normalized collision energy of 40% for HCD fragmentation, and dynamic exclusion applied for 60 s periods. Charge-state screening was enabled to reject unassigned and singly charged protonated ions.

### 4.7. Quantitative Proteomic Data Analysis and Representation

Peptide identification from raw data was carried out using PEAKS Studio X+ search engine (Bioinformatics Solutions Inc., Waterloo, ON, Canada). Database search was performed against uniprot-mus-musculus.fasta (55,462 entries; UniProt release 03/2020) merged to uniprot-trypanosoma-cruzi (19,242 entries; UniProt release 03/2020). The following constraints were used for the searches: tryptic cleavage after Arg and Lys (semi-specific), up to two missed cleavage sites, and tolerances of 20 ppm for precursor ions and 0.05 Da for MS/MS fragment ions, and the searches were performed allowing optional Met oxidation and Cys carbamidomethylation and fixed TMT 6plex reagent labeling at the N-terminus and lysine residues. FDR for peptide spectrum matches and proteins was limited to 0.01. Only those proteins with at least two unique peptides discovered from LC/MS/MS analyses were considered reliably identified and sent to be quantified [63,64,65].

Quantitation of TMT labeled peptides was performed with PEAKS Studio X+ search engine, selected “Reporter Ion Quantification iTRAQ/TMT” under the “Quantifications” options. We used auto-normalization mode, which calculates a global ratio from the total intensity of all labels in all quantifiable peptides. The −10LgP, quality, and reporter ion intensity were used for the spectrum filter, and significance (PEAKSQ method) was used for peptide and protein abundance calculation. For the protein quantification, we considered protein groups for peptide uniqueness, used unique peptides for protein quantification, and the modified peptides were excluded.

For data representation, volcano plots and bar plots of each analysis were created using R programming language (v.4.2.2) with the package ggplot2. Enrichment GO terms study was carried out with Enrichr webtool, posterior REVIGO [66] analysis, and represented by Cytoscape (v.3.10.1). Protein–protein interaction networks were performed by STRING webtool (v.12.0) and represented by Cytoscape (version 3.10.1). Enrichment pathways analysis was performed in the Enrichr web tool using the BioPlanet database. Venn diagrams were created with the following online tool: https://bioinformatics.psb.ugent.be/webtools/Venn/ accessed on 4 July 2024. Figure representations were prepared in Adobe Illustrator CS5 (2010).

## Figures and Tables

**Table 1:** Most remarkable upregulated or downregulated macrophage pathways in our proteomic analysis. Text color legend for pathways shared by all the conditions (red); three conditions (green); two conditions (blue); unique to a condition (black). Abbreviations: LCFA, long-chain fatty acid; ALA, alpha-linolenic acid; LA, linoleic acid; GSL, glycosphingolipid; Cer, ceramide; GL, glycolipid; AEA, anandamide; PG, phosphatidylglycerol; GP, glycoprotein; GAG, glycosaminoglycan; OS, oligosaccharide; ERAD, endoplasmic-reticulum-associated protein degradation; PKC, protein kinase C; RANKL, receptor activator of nuclear factor kappa-B ligand; TWEAK, TNF-related weak inducer of apoptosis. Arrows up indicate upregulation and arrows down indicate downregulation.

	BALB/c Y	BALB/c VFRA	*Slamf1^-/-^* Y	*Slamf1^-/-^* VFRA
Interferons	↑ IFN type I-II responses	↑ IFN type I-II responses	↑ IFN type I-II responses	↑ IFN type I-II responses
TLRs	↓ TLR2/TLR4 signaling	↓ TLR2/TLR4 signaling	↓ TLR2/TLR4 signaling	↓ TLR2/TLR4 signaling
	↑ TLR7/TLR9 signaling		↑TLR7/TLR9 signaling	
Interleukins	↑ IL-18 production	↑ IL-18 production	↑ IL-18 production	↑ IL-18 production
	↑ IL-2 signaling		↑ IL-2 signaling	↑ IL-2 signaling
		↑ IL-11 signaling		↑ IL-11 signaling
Lipid metabolism	↑ LCFA import	↑ LCFA import	↑ LCFA import	↑ LCFA import
	↑ ALA metabolism	↑ ALA metabolism	↑ ALA metabolism	↑ ALA metabolism
	↑ LA metabolism	↑ LA metabolism	↑ LA metabolism	↑ LA metabolism
	↓ GSL biosynthesis	↓ GSL biosynthesis		↓ GSL biosynthesis
	↓ Cer and GL transport	↓ GSL catabolism		↓ GSL catabolism
		↓ AEA metabolism		↓ AEA metabolism
		↓ PG biosynthesis		↓ PG biosynthesis
Carbohydrate metabolism	↓ GP catabolism	↓ GP catabolism	↓ GP catabolism	↓ GP catabolism
	↓ GAG degradation	↓ GAG degradation		↓ GAG degradation
		↓ OS catabolism	↓ OS catabolism	↓ OS catabolism
		↓ protein deglycosylation	↓ protein deglycosylation	↓ protein deglycosylation
Effects on other immune cells	↑ αβ T cell activation		↑ αβ T cell activation	
	↓ neutrophil activation	↓ neutrophil activation		
Phagocytic processes			↓ vacuolar acidification	
			↓ clathrin-derived vesicle budding	
			↓ lysosome biogenesis	
			↓ phagosome processes	
Other intracellular pathways	↑ NLRP3 inflammasome	↑ NLRP3 inflammasome	↑ NLRP3 inflammasome	↑ NLRP3 inflammasome
		↓ ERAD pathway	↓ ERAD pathway	↓ ERAD pathway
	↑ protein translation	↑ pexophagy/aggrephagy	↑ integrin activation	
	↓ aggrephagy	↑ β2 integrin pathway	↑ PKC signaling	
		↑ RANKL pathway	↓ iron uptake & transport	
		↑ TWEAK regulation		

## Data Availability

Data are contained within the article and Supplementary Materials.

## Supplementary Materials

The following supporting information can be downloaded at: https://www.mdpi.com/article/10.3390/ijms25137493/s1.
