# Supplementary material for: Quantitative Proteomic Analysis of Macrophages Infected with Trypanosoma cruzi Reveals Different Responses Dependent on the SLAMF1 Receptor and the Parasite Strain
Source: Int J Mol Sci. 2024 Jul 8;25(13):7493. doi: 10.3390/ijms25137493 (PMC11242706; doi:10.3390/ijms25137493)
Supplement: Supplementary file 1 [file ijms-25-07493-s001.zip › Supplementary Tables.pdf]

Supplementary Tables

Table S1. Enriched upregulated GO terms shared between BALB/c and *Slamf1*$^{-/-}$ macrophages infected by Y and VFRA strains.

Conditions	Number	GO terms
BALB/c VFRA BALB/c Y *Slamf1*$^{-/-}$ VFRA *Slamf1*$^{-/-}$ Y	11	positive regulation of IL-18 production<br>long-chain fatty acid import into cell<br>positive regulation of cellular extravasation<br>regulation of long-chain fatty acid import across plasma membrane<br>regulation of leukocyte mediated cytotoxicity<br>receptor-mediated virion attachment to host cell<br>negative regulation of TLR4 signaling pathway<br>positive regulation of humoral immune response<br>negative regulation of TLR2 signaling pathway<br>lipid import into cell<br>positive regulation of NLRP3 inflammasome complex assembly
BALB/c VFRA BALB/c Y *Slamf1*$^{-/-}$ Y	1	cellular response to type II IFN
BALB/c Y *Slamf1*$^{-/-}$ VFRA *Slamf1*$^{-/-}$ Y	4	defense response to virus<br>positive regulation of nitric oxide synthase biosynthetic process<br>defense response to symbiont<br>NAD biosynthesis via nicotinamide riboside salvage pathway
BALB/c VFRA BALB/c Y *Slamf1*$^{-/-}$ VFRA	4	positive regulation of response to external stimulus<br>positive regulation of transmembrane transport<br>positive regulation of response to biotic stimulus<br>alpha-linolenic acid metabolic process
BALB/c VFRA *Slamf1*$^{-/-}$ VFRA *Slamf1*$^{-/-}$ Y	1	response to IFN-β
BALB/c Y *Slamf1*$^{-/-}$ Y	5	positive regulation of TLR7 and TLR9 signaling pathway<br>alpha-beta T cell activation<br>regulation of T-helper 2 cell cytokine production<br>regulation of viral genome replication<br>type I IFN-mediated signaling pathway
BALB/c Y *Slamf1*$^{-/-}$ VFRA	1	adipose tissue development
BALB/c VFRA *Slamf1*$^{-/-}$ Y	1	cellular response to IL-1
BALB/c VFRA *Slamf1*$^{-/-}$ VFRA	4	positive regulation of cytokine production involved in inflammatory response<br>response to type II IFN<br>cellular response to steroid hormone stimulus<br>regulation of cell killing

Table S2. Unique enriched upregulated GO terms in BALB/c and *Slamf1*$^{-/-}$ macrophages infected by Y and VFRA strains.

Conditions	Number	GO terms
BALB/c Y	11	ribosome biogenesis<br>rRNA metabolic process<br>protein targeting to ER cotranslational<br>protein targeting to membrane<br>regulation of helicase activity<br>cellular response to exogenous dsRNA<br>cytoplasmic translation

		nuclear-transcribed mRNA catabolic process, nonsense-mediated decay<br>regulation by virus of viral protein levels in host cell<br>positive regulation of immune response<br>response to type I interferon
Slamf1$^{-/-}$ Y	18	positive regulation of cell activation<br>membrane fission<br>protein poly-ADP-ribosylation<br>regulation of immune response<br>regulation of chemokine (C-C motif) ligand 5 production<br>regulation of nuclease activity<br>ubiquitin-independent protein catabolic process via the multivesicular body sorting pathway<br>response to tumor necrosis factor<br>protein localization to organelle<br>positive regulation of protein-containing complex assembly<br>regulation of ribonuclease activity<br>regulation of calcium-mediated signaling<br>regulation of inositol phosphate biosynthetic process<br>positive regulation of integrin activation<br>positive regulation of chromatin binding<br>cytokine-mediated signaling pathway<br>regulation of innate immune response<br>protein kinase C signaling
BALB/c VFRA	12	protein localization to perinuclear region of cytoplasm<br>endothelial cell development<br>pexophagy<br>aggrephagy<br>long-chain fatty-acyl-CoA metabolic process<br>autophagy of peroxisome<br>very long-chain fatty acid metabolic process<br>leukocyte cell-cell adhesion<br>T cell activation involved in immune response<br>negative regulation of endothelial cell apoptotic process<br>response to mitochondrial depolarisation<br>protein localization to Golgi apparatus
Slamf1$^{-/-}$ VFRA	4	response to interferon-alpha<br>long-chain fatty-acyl-CoA biosynthetic process<br>negative regulation of viral life cycle<br>positive regulation of leukocyte proliferation

Table S3. Enriched downregulated GO terms shared between BALB/c and *Slamf1*$^{-/-}$ macrophages infected by Y and VFRA strains.

Conditions	Number	GO terms
BALB/c VFRA<br>BALB/c Y *Slamf1*$^{-/-}$<br>VFRA *Slamf1*$^{-/-}$ Y	4	regulation of endothelial cell-matrix adhesion via fibronectin<br>glycoprotein catabolic process<br>wound healing, spreading of epidermal cells<br>positive regulation of epithelial cell proliferation involved in wound healing
BALB/c VFRA<br>BALB/c Y *Slamf1*$^{-/-}$<br>VFRA	1	keratan sulfate catabolic process
BALB/c VFRA<br>*Slamf1*$^{-/-}$ VFRA<br>*Slamf1*$^{-/-}$ Y	2	oligosaccharide catabolic process<br>protein deglycosylation
BALB/c VFRA<br>BALB/c Y	2	neutrophil activation involved in immune response<br>neutrophil mediated immunity

BALB/c Y *Slamf1*$^{-/-}$ VFRA	3	cellular response to muramyl dipeptide<br>positive regulation of collagen biosynthetic process<br>intermediate filament organization
BALB/c VFRA *Slamf1*$^{-/-}$ Y	2	negative regulation of mitochondrial fusion<br>regulation of isotype switching
BALB/c VFRA *Slamf1*$^{-/-}$ VFRA	2	striated muscle cell differentiation<br>glycosphingolipid catabolic process

Table S4. Unique enriched downregulated GO terms in BALB/c and *Slamf1*$^{-/-}$ macrophages infected by Y and VFRA strains.

Conditions	Number	GO terms
BALB/c Y	4	ceramide transport<br>glycolipid metabolic process<br>aggrephagy<br>glycolipid transport
Slamf1$^{-/-}$ Y	5	iron ion transport<br>intracellular protein transport<br>vacuolar acidification<br>DNA catabolic process<br>apoptotic nuclear changes
BALB/c VFRA	1	cellular response to xenobiotic stimulus
Slamf1$^{-/-}$ VFRA	2	vesicle transport along actin filament<br>glomerular filtration
