# Supplementary figures and images for: Quantitative Proteomic Analysis of Macrophages Infected with Trypanosoma cruzi Reveals Different Responses Dependent on the SLAMF1 Receptor and the Parasite Strain
Source: Int J Mol Sci. 2024 Jul 8;25(13):7493. doi: 10.3390/ijms25137493 (PMC11242706; doi:10.3390/ijms25137493)

A

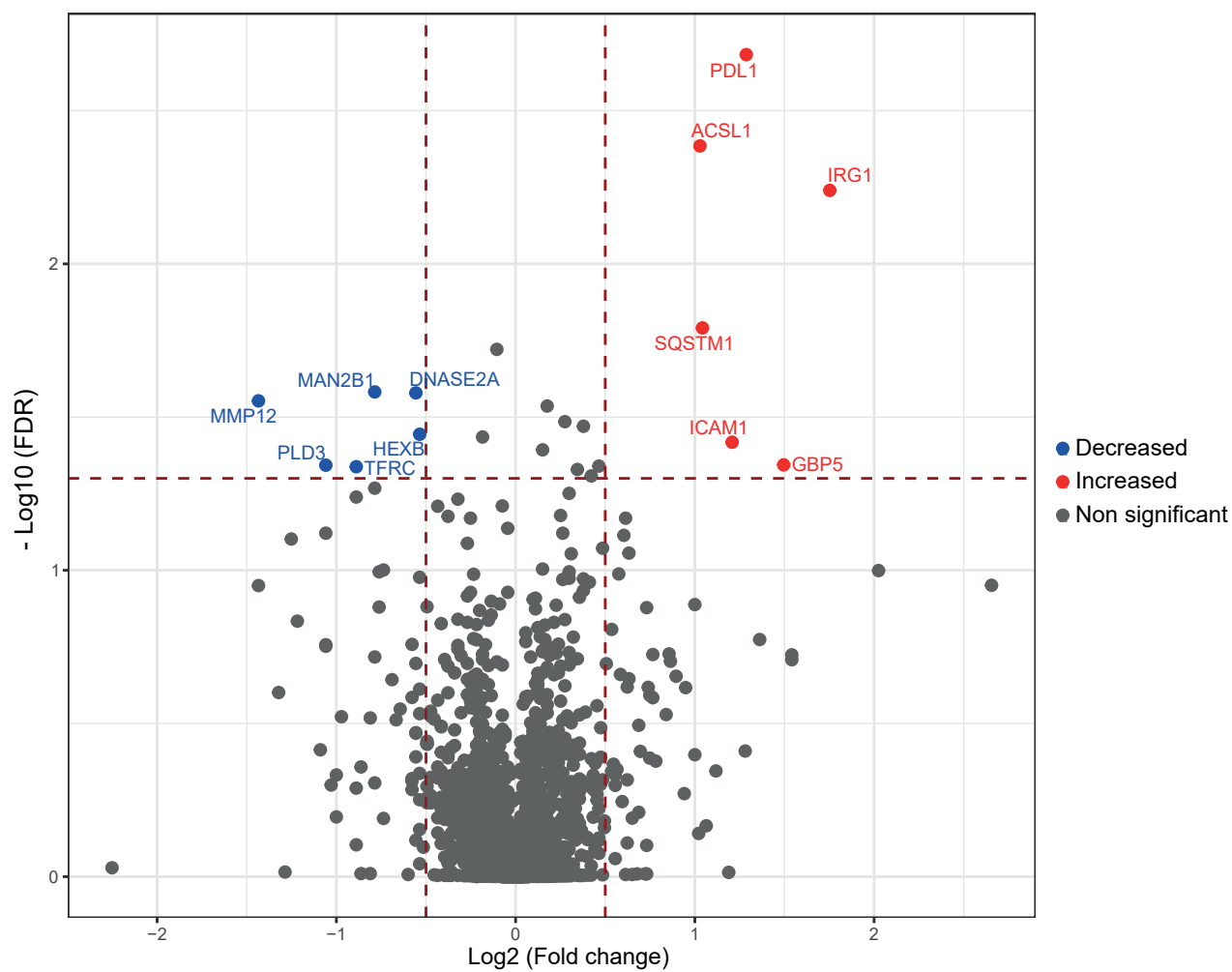

B

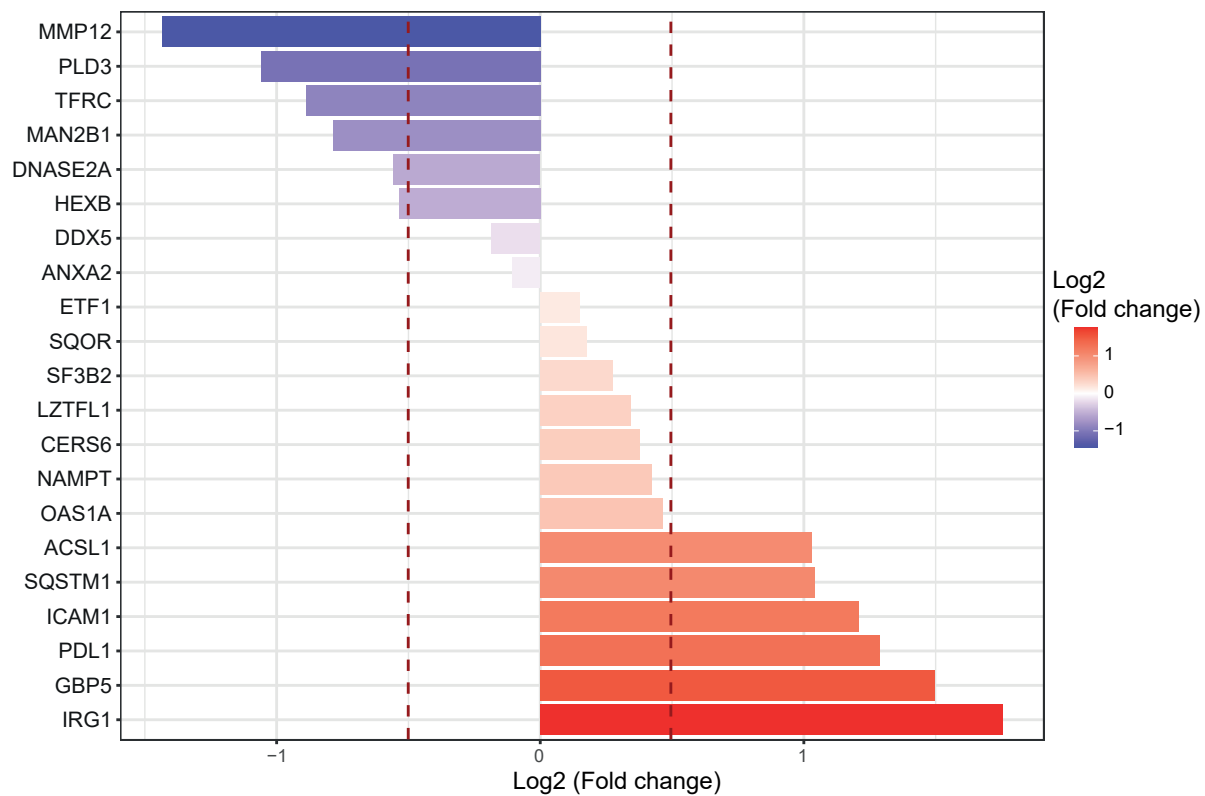

Supplement: Supplementary file 1 [file ijms-25-07493-s001.zip › Supplementary_Figure_S1.pdf]

A

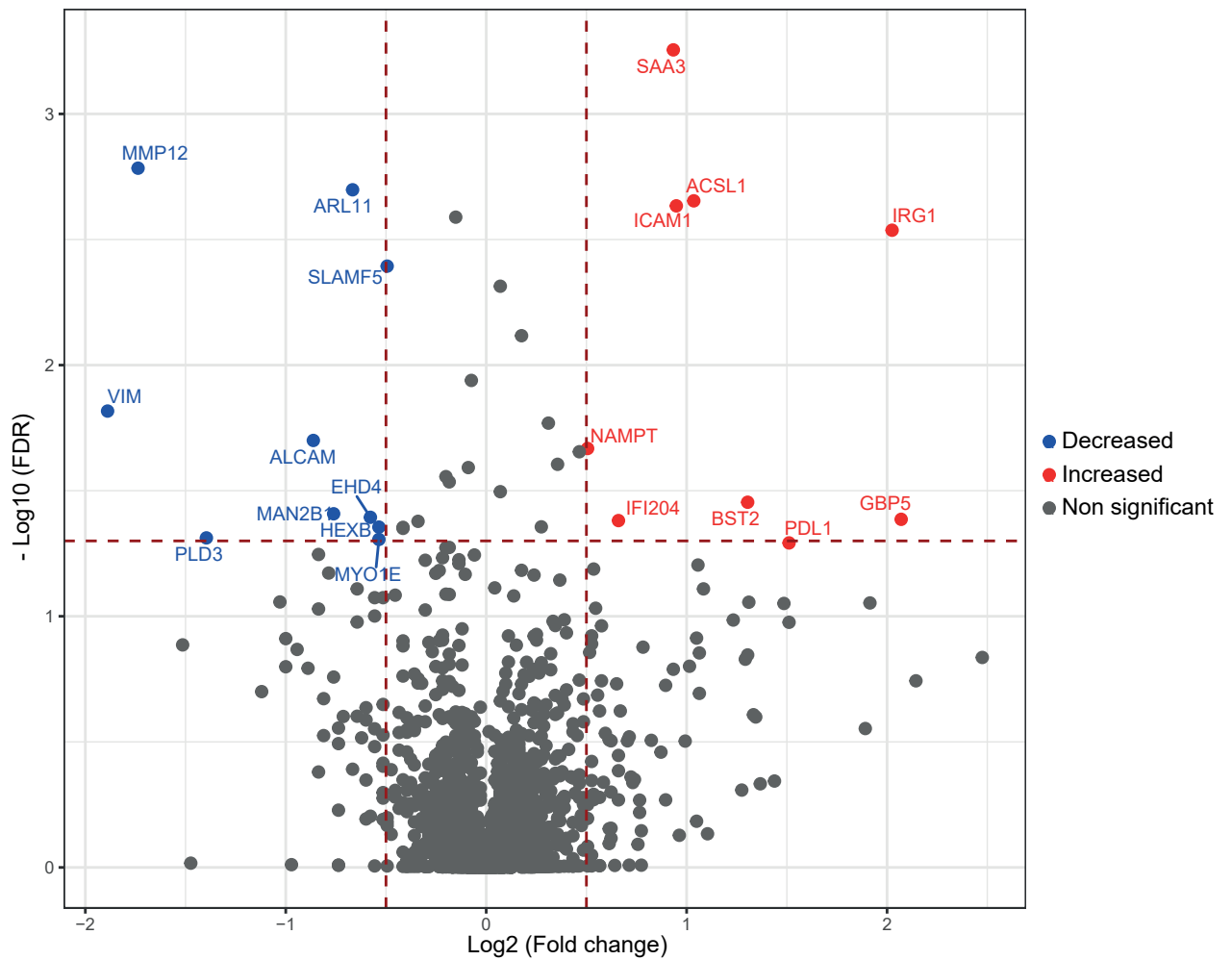

B

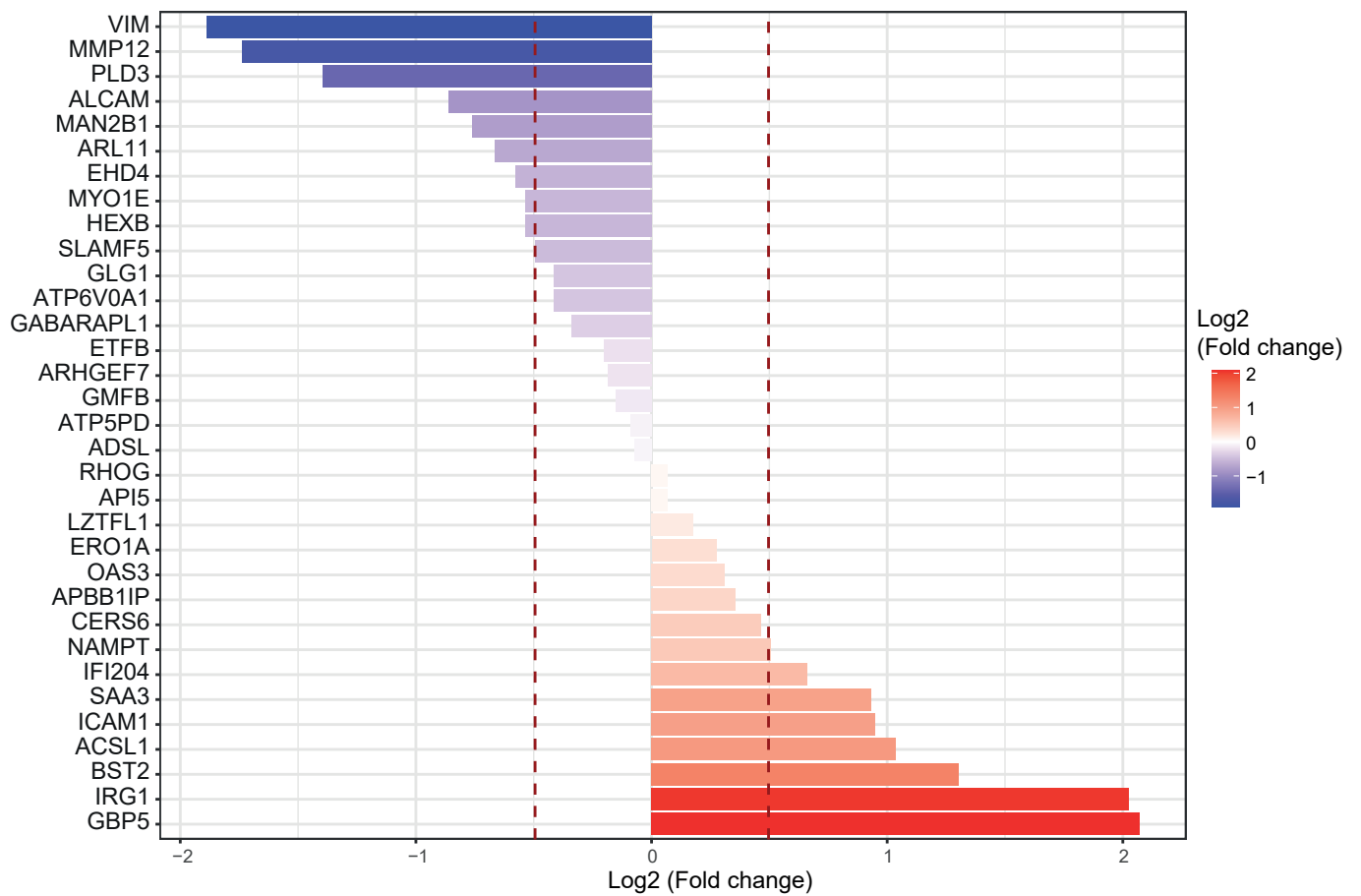

Supplement: Supplementary file 1 [file ijms-25-07493-s001.zip › Supplementary_Figure_S2.pdf]

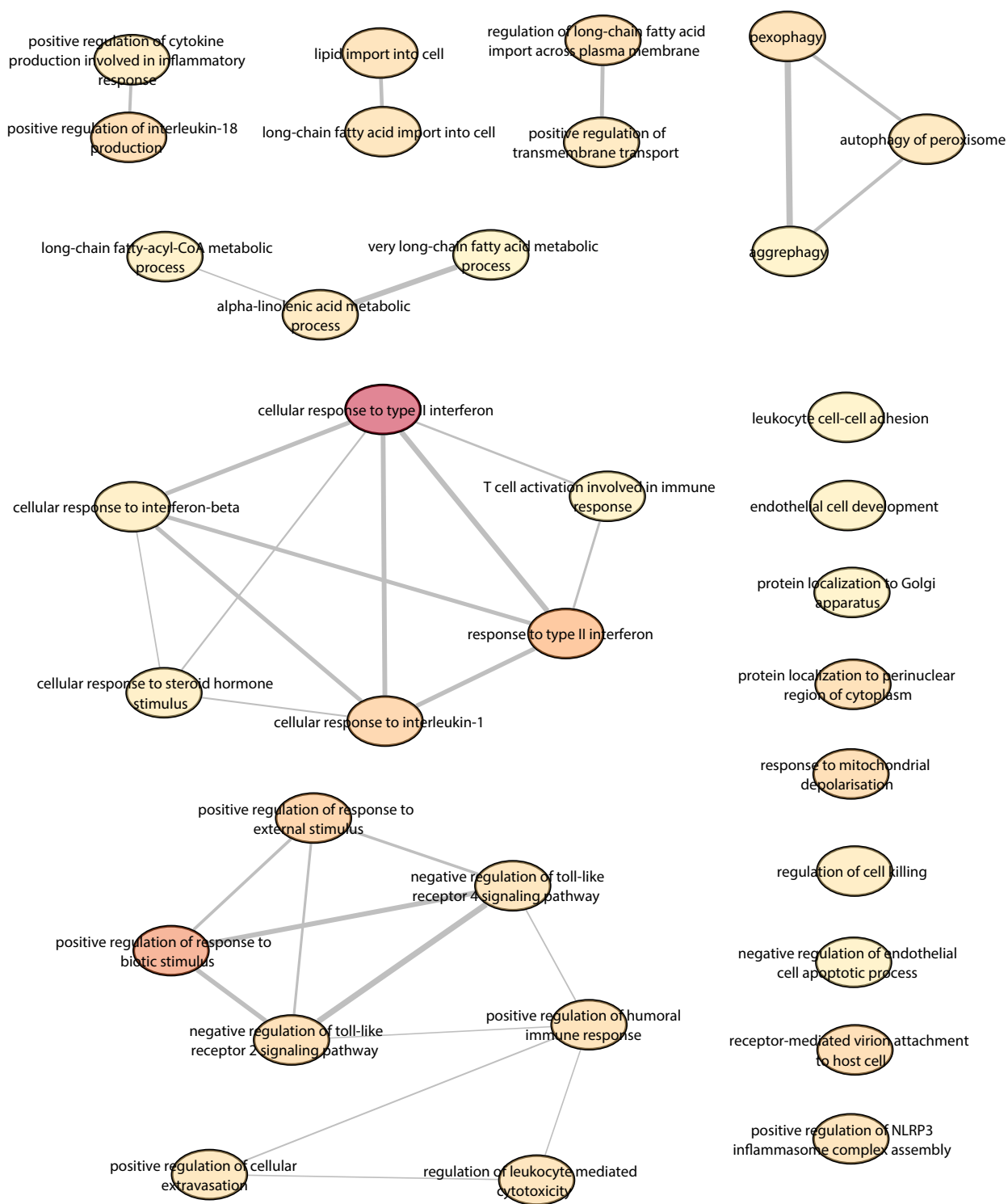

Log10 (*p*-value)

Node Fill Color: value

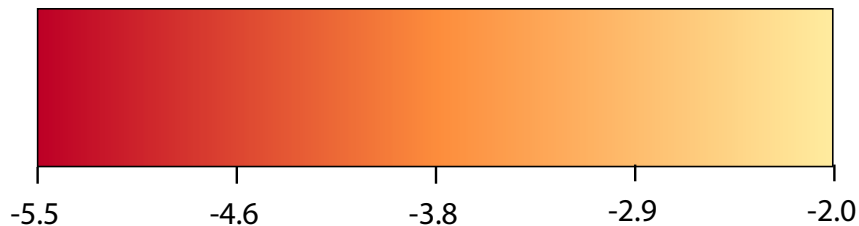

Supplement: Supplementary file 1 [file ijms-25-07493-s001.zip › Supplementary_Figure_S3.pdf]

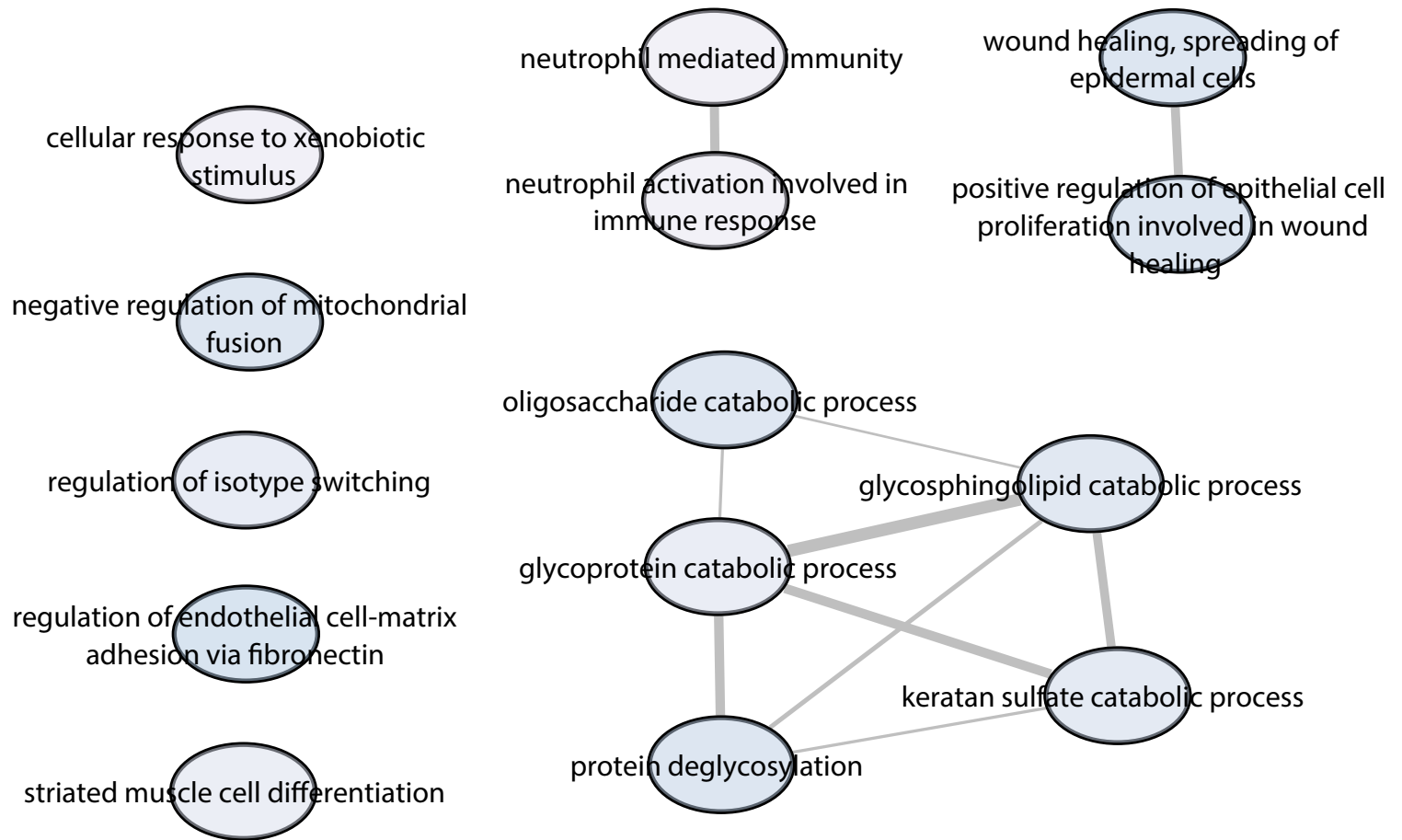

$\text{Log}_{10}(p\text{-value})$

Node Fill Color: value

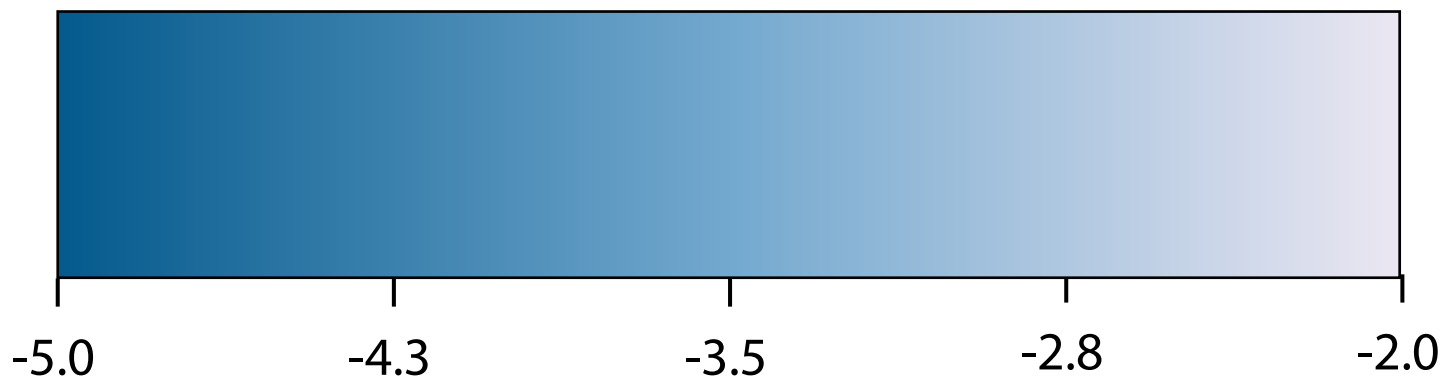

Supplement: Supplementary file 1 [file ijms-25-07493-s001.zip › Supplementary_Figure_S4.pdf]

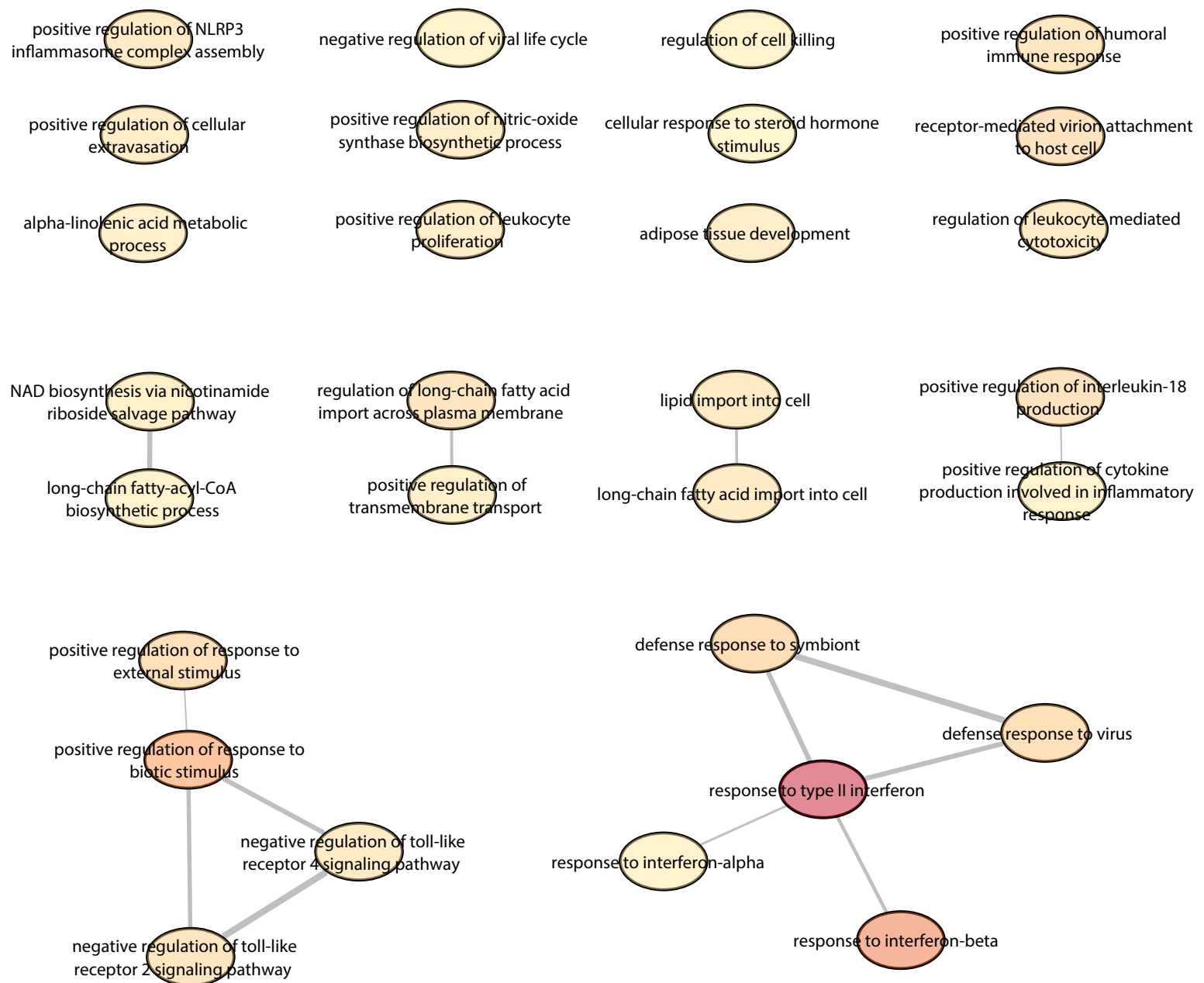

Log10 (*p*-value)

Node Fill Color: value

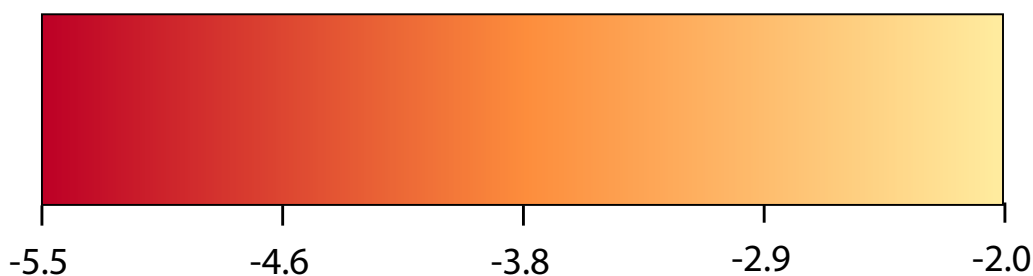

Supplement: Supplementary file 1 [file ijms-25-07493-s001.zip › Supplementary_Figure_S5.pdf]

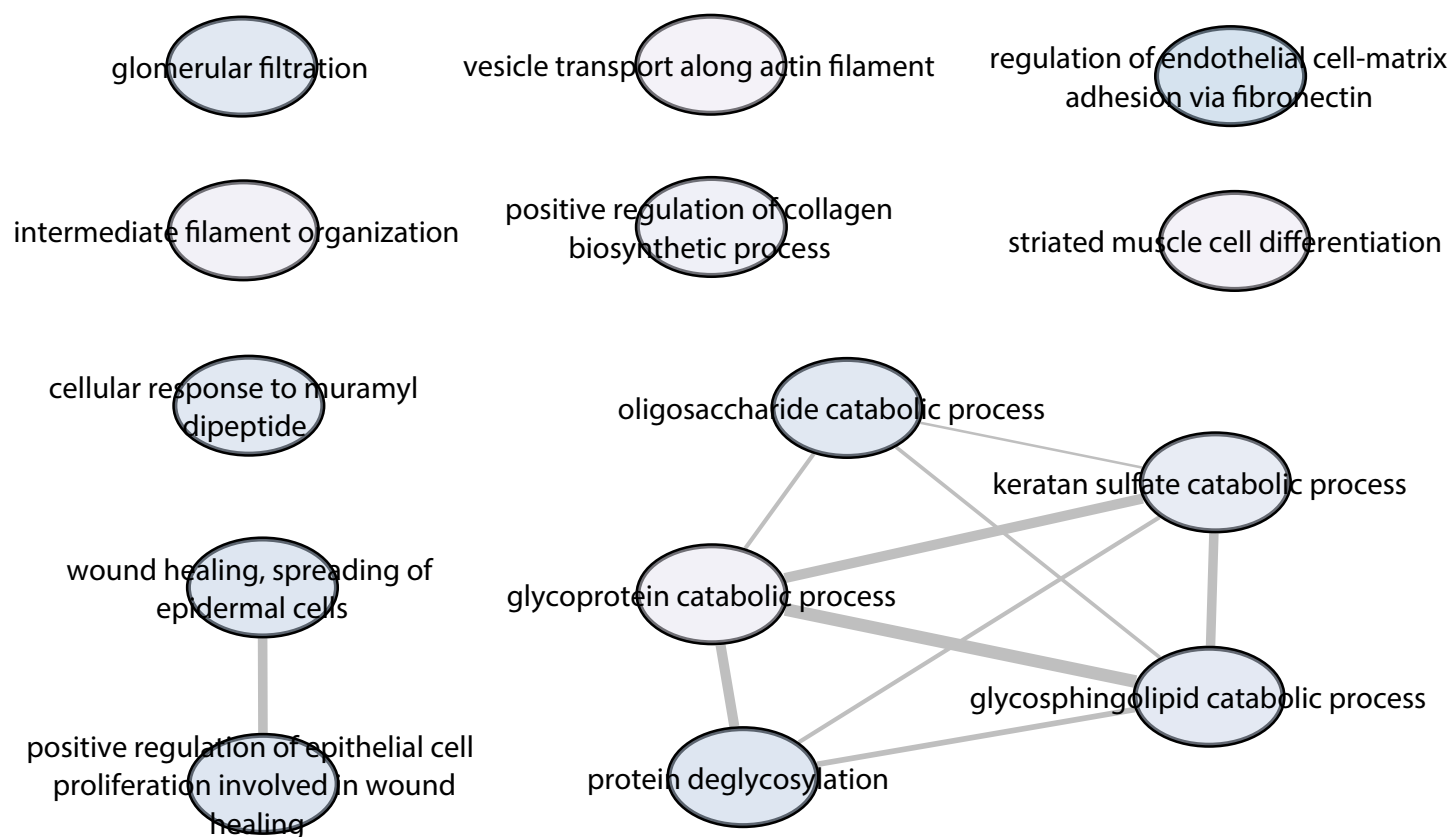

$\text{Log}_{10}(p\text{-value})$

Node Fill Color: value

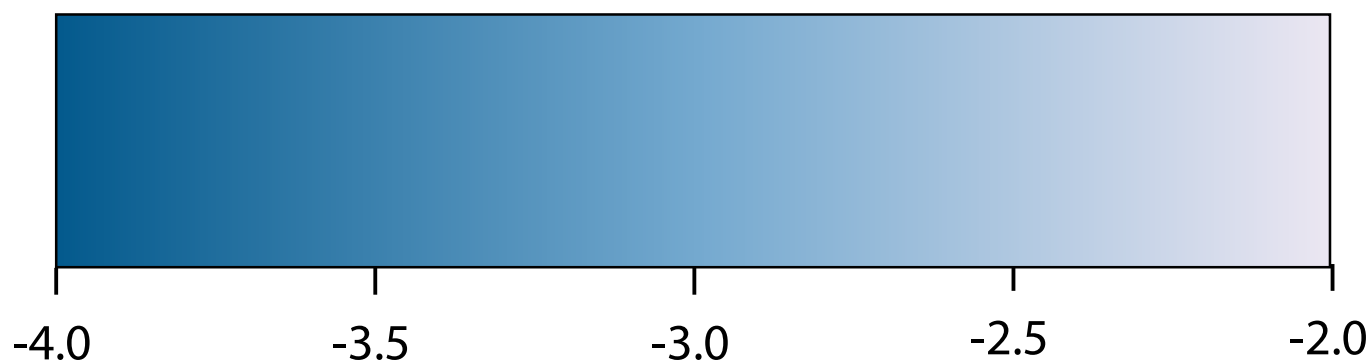

Supplement: Supplementary file 1 [file ijms-25-07493-s001.zip › Supplementary_Figure_S6.pdf]

A

BALB/c Y

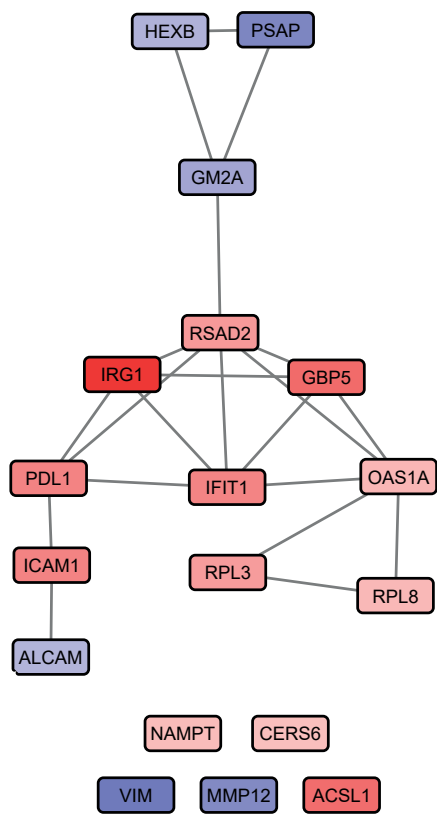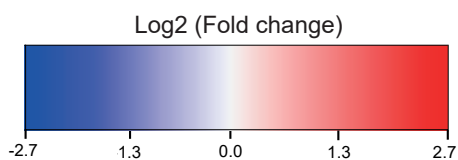

B

*Slamf1*<sup>-/-</sup> Y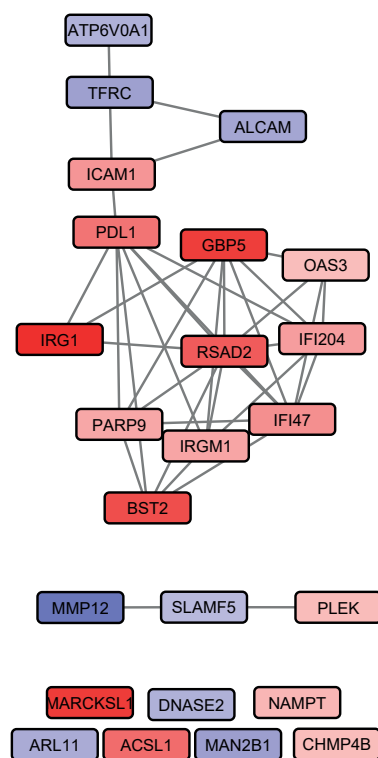

C

BALB/c VFRA

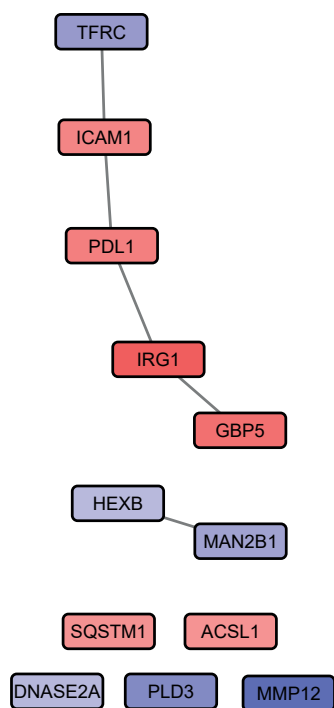

D

*Slamf1*<sup>-/-</sup> VFRA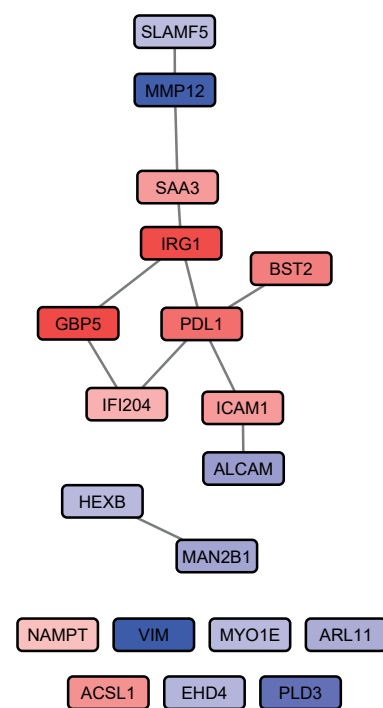

Supplement: Supplementary file 1 [file ijms-25-07493-s001.zip › Supplementary_Figure_S7.pdf]
